# Pre- and Postnatal Vitamin D Status and Allergy Outcomes in Early Childhood

**DOI:** 10.3390/biomedicines10050933

**Published:** 2022-04-19

**Authors:** Kristina Rueter, Aris Siafarikas, Debra J. Palmer, Susan L. Prescott

**Affiliations:** 1 School of Medicine, The University of Western Australia, 35 Stirling Highway, Crawley 6009, Australia; aris.siafarikas@health.wa.gov.au (A.S.); debbie.palmer@uwa.edu.au (D.J.P.); susan.prescott@telethonkids.org.au (S.L.P.); 2 Department of Immunology, Perth Children’s Hospital, 15 Hospital Avenue, Nedlands 6009, Australia; 3 inVIVO Planetary Health, Group of the Worldwide Universities Network (WUN), 6010 Park Ave, West New York, NJ 07093, USA; 4 Telethon Kids Institute, The University of Western Australia, 15 Hospital Avenue, Nedlands 6009, Australia; 5 Department of Endocrinology, Perth Children’s Hospital, 15 Hospital Avenue, Nedlands 6009, Australia; 6 Institute for Health Research, University of Notre Dame, Fremantle 6160, Australia

**Keywords:** vitamin D, non-communicable disease, allergic disease, pregnancy, infancy, early childhood, environmental factors, immunomodulatory functions, vitamin D observational trials, vitamin D randomized controlled trials

## Abstract

The dramatic increase in the prevalence of allergic disease in recent decades reflects environmental and behavioural changes that have altered patterns of early immune development. The very early onset of allergic diseases points to the specific vulnerability of the developing immune system to environmental changes and the development of primary intervention strategies is crucial to address this unparalleled burden. Vitamin D is known to have immunomodulatory functions. While allergic disease is multifactorial, associations with reduced sunlight exposure have led to the hypothesis that suboptimal vitamin D levels during critical early periods may be one possible explanation. Interventions to improve vitamin D status, especially in early life, may be the key to allergic disease prevention.

## 1. Introduction

The prevalence of non-communicable diseases (NCDs)—including allergic disease as a very early presentation—has increased dramatically in recent decades. This trend, described as “the epidemic of the 21st century”, recognizes allergy as a major cause of morbidity in the developed world without any evidence of this crisis abating [1,2,3]. Although genetic risk factors influence individual susceptibility to disease, the rise in allergic diseases and other NCDs in recent decades has been far too rapid to be accounted for by changes in the population gene pool alone [4]. 

When looking for potential factors driving the increasing risk of allergic diseases, the dramatic environmental and lifestyle changes of the modern age are clearly implicated [5]. With economic prosperity and associated lifestyle changes, the rising propensity for inflammation is implicated in the rising burden of chronic disease including allergic diseases in all modern societies. It is therefore critically important to understand the normal processes and pathways that underpin immune development, how these are modified by adverse early environmental exposures, and how these may be favorably modified to reduce disease risk.

**Figure 1:**
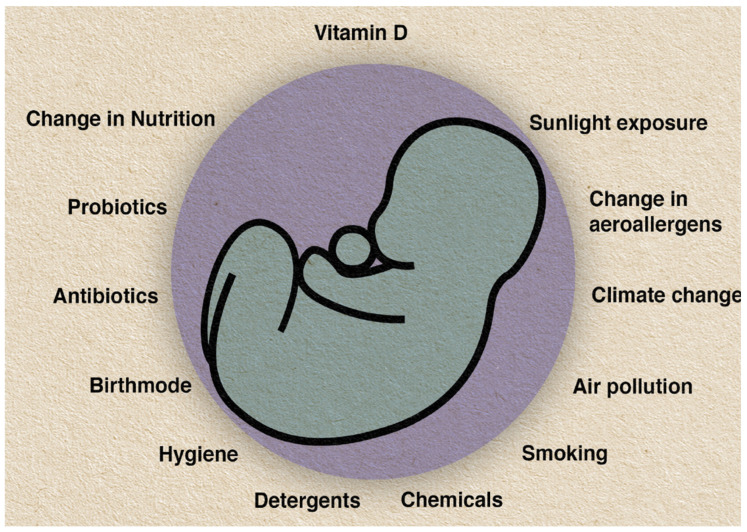
A number of potentially causative pre- and early postnatal environmental factors may influence the development of allergic diseases. The hygiene hypothesis suggests that increased hygiene with a lack of exposure to microbes and parasitic infections at an early age prevents the developing immune system from forming tolerance. In modern societies, increased time is spent indoors, which can lead to reduced sunlight exposure and vitamin D production. Additionally, change in nutrition and use of pro- and antibiotics, detergents and chemicals need to be considered in the context of allergy development. Furthermore, mode of delivery, smoking, air pollution, climate change and change in aeroallergens may contribute to the development of allergic diseases in early childhood.

Whilst the impact of modern environments on the immune system is likely to be complex and multifaceted, declining microbiome biodiversity (particularly in commensal microflora), exposure to air pollution, cigarette smoke and ingested environmental contaminants [6,7], chemical allergens and irritants from cosmetic products [8], medications, increased time spent indoors and dietary changes associated vitamin D deficiency [9] have emerged as potential causative factors influencing the development of allergic diseases. Here, timing plays a significant role. Prenatally, and during infancy, childhood and adolescence, the human body is in a constant change of development. Thus, the predefined stages of maturation display windows of accessibility, vulnerability and opportunity, where health intervention and prevention can present themselves in interactions between genes, environment, and critical developmental time periods. There is a need to intervene early to prevent disease in the first instance, by targeting the modifiable environmental risk factors that are most likely responsible [10,11], which is illustrated in Figure 1 showing potential influencing prenatal and early postnatal environmental factors.

As one key factor under consideration (Figure 1), as both a causal factor and a possible allergy prevention strategy, this review article will focus on vitamin D—important in this context because of known immunomodulatory functions, including immune pathways relevant for protecting against allergic diseases. Vitamin D deficiency and insufficiency are global phenomena afflicting more than one billion people in all age groups. As over 90% of natural vitamin D production is dependent on ultraviolet radiation (UVR), declining exposure to UVR with increasingly indoor-centered modern lifestyles is implicated in adverse vitamin D status and has been connected with the rising rates of immune and inflammatory diseases in many parts of the world. Although the physiological effects of this are likely to have a broad impact on many aspects of health, this review will focus particularly on the implications for the possible prevention of allergic diseases. 

Vitamin D is known to have immunomodulatory functions and considering that immune mechanisms underly all allergic diseases, this might be a factor which beneficially modulates immune function to prevent allergies. So far, numerous observational trials and a few intervention trials have been performed, with conflicting results. In the context of reviewing further literature for vitamin D as a modifiable environmental risk factor, it is useful to first briefly describe sources of vitamin D, followed by vitamin D metabolism and immunomodulatory functions, before further exploring clinical trials on the influence of vitamin D on allergy outcomes.

## 2. Environmental Factors as Causes for the Global Rise of Vitamin D Deficiency and Sources of Vitamin D

Considering that up to 90 to 95% of vitamin D can be provided by cutaneous synthesis under the influence of UVB light [12], the major cause for the vitamin D deficiency pandemic is a lack of appreciation that UV light exposure is the major physiological source of vitamin D [13,14,15]. Increased time spent indoors due to the exponential rise of indoor employment and relaxation activities (particularly an increase in screen time) in modern times as well as sun protection behaviours [16,17,18] are lifestyle factors which have an influence on UV light exposure. In addition, lower UVR levels are found in high-latitude locations and winter season. Furthermore spending time outdoors exclusively in the morning or after 3 p.m. leads to reduced UV light expsosure [19].

Considering physiological factors, increased skin pigmentation [20,21], aging and obesity have an influence on vitamin D status [17]. Low dietary vitamin D intakes caused by changes in nutrition from more traditional vitamin D-containing food sources to modern commercial, highly processed fast food can contribute to vitamin D deficiency by destroying the naturally contained vitamin D. 

Vitamin D occurs in two forms: vitamin D2 and vitamin D3. Vitamin D2 is obtained from the UV irradiation of the yeast sterol ergosterol and is found in sun-exposed and sun-dried mushrooms. Vitamin D3 is found in oil-rich fish such as herring, salmon and mackerel, tuna, cod liver oil and egg yolk [12]. People living in the far southern or northern latitudes may also obtain vitamin D from seal blubber, whales or polar bear liver [14]. In addition, foods enriched with fortified vitamin D [12] and nutritional supplements play a role in maintaining a sufficient vitamin D status. 

## 3. Vitamin D Metabolism and Physiology 

Vitamin D is a pre-hormone which historically plays a critical role in calcium and mineral homeostasis, bone modeling and remodeling. However, the role of vitamin D goes far beyond this, as different activities of vitamin D ensure proper functioning of vital human organs including the skin [22]. Apart from functioning *on* the skin, vitamin D is also predominantly derived *from* the skin via sun exposure, which makes vitamin D unique among hormones [12,23,24].

During sun exposure, 7-dehydrocholesterol in the skin absorbs UVB radiation and undergoes chemical rearrangements to form the thermodynamically stable vitamin D3 (reviewed in [19]). Vitamin D2 and D3 act as pro-hormones and, when activated, have the same biological activity. Following ingestion or transport from the skin to the circulation, these calciferols are bound to the vitamin D-binding protein (DBP) [25]. DBP transports the calciferols to the liver, where the first step of hydroxylation to 25-hydroxyvitamin D (25(OH)D) takes place (reviewed in [26]).

While 25(OH)D is the predominant circulating metabolite, it is largely inert and displays minimal binding affinity to the vitamin D receptor (VDR) [26]. Hence, to gain biological activity in a second step, 25(OH)D must undergo further renal hydroxylation [26]. 1,25(OH)_2_D (calcitriol) is the active metabolite of vitamin D which is bound to DBP while circulating in the blood and transported to the VDR. After transport to the VDR, 1,25(OH)_2_D is released to dock on the VDR and induce processes in the cell nucleus [12,27]. However, 1,25(OH)_2_D is not used as a measure of vitamin D status [28] due to its short circulating half-life time (4 h), low serum concentrations and tight regulation [28]. Instead, 25(OH)D is used to evaluate vitamin D status as it has a long half-life time (2–3 weeks), provides stable serum concentrations and is present at concentrations approximately 1000-fold greater than 1,25(OH)_2_D [28]. The international Endocrine Society’s guidelines define vitamin D deficiency as 25(OH)D < 50 nmol/L (<20 ng/mL), and vitamin D insufficiency as <75 nmol/L (<30 ng/mL) [28].

More recent research revealed that calcitriol modulates activation, proliferation and differentiation of immune and inflammatory cells through the VDR expressed on these cells [29,30,31,32,33]. Apart from immune cells [33], the VDR is also present on cells of the skin [34] and cardiovascular system [35], and these functions may provide some explanation for the epidemiological associations between vitamin D status and NCDs including cardiometabolic conditions, malignancies and immune disorders [36]. Hence, the next section will further explore the role of the VDR associated with this DBP.

## 4. Vitamin D Receptor and Vitamin D-Binding Protein Polymorphisms and Links to Allergic Diseases

The VDR is a member of the nuclear receptor family of transcription factors and is found on most cells of the human body including skin cells (basal layer of the epidermis, keratinocytes and hair follicles) [37] and immune cells, with effects on cell differentiation, proliferation and apoptosis [38]. It is therefore quite feasible that VDR polymorphisms on interaction with environmental factors could significantly influence immune regulation by altering cell proliferation and differentiation [39]. Genetic studies have provided early evidence of a potential role of the VDR in the genesis of allergic diseases. Looking at the link to asthma specifically, the association between a number of VDR restriction fragment length polymorphisms and the risk of asthma has been described [40]. However, other studies could not confirm the role of VDR polymorphisms on allergy outcome [41,42]. In a recent review article, Tamasauskiene et al. conclude that the association of VDR gene polymorphisms and vitamin D with asthma, allergic rhinitis and atopy is variable [43]. The inconsistency between findings may be due to different study designs, but also likely reflects that allergic diseases are complex, involving multiple genetic and environmental factors [40,43].

There are numerous other genes involved in the vitamin D metabolism pathway that have been associated with the risk of allergic disease [44,45,46]. DBP polymorphisms and vitamin D deficiency may jointly or independently contribute to a variety of skeletal and non-skeletal adverse health outcomes including different NCDs such as osteoporosis, diabetes, thyroid autoimmunity, inflammatory bowel disease, chronic lung disease (reviewed in [47]) and allergy development [48,49]. The exact role of DBP in the pathophysiology of all these inflammatory diseases is, however, not completely understood. Additionally, as in other genetic associations, the influence of DBP on allergic diseases is likely to be affected by gene–gene and gene–environment interactions and we will have to await further studies to investigate these complex associations.

After giving an overview on vitamin D pathophysiology, metabolism and associated nuclear actions and polymorphisms, this review will now turn to some of the potential immune-modulating properties by which vitamin D may alter the course of allergic disease. An understanding of the potential immunological mechanisms is crucial as this explains clinical findings. 

## 5. Immunoregulatory Functions of Vitamin D and the Influence on Allergic Disease Development

A link between vitamin D and the immune system was first acknowledged approximately four decades ago with the expression of the VDR in both activated T and B cells [33]. Since then, the body of evidence suggesting that vitamin D, especially its active metabolite, plays a key role in modulating the physiological activity of the immune system has grown. 

### 5.1. The Role of Vitamin D in the Th1/Th2 Dichotomy 

Early experiments showed that the active metabolite 1,25(OH_2_)D_3_ inhibits interleukin (IL)-2 production and T-cell proliferation [33,50]. Th (T-helper) cells are a primary target for 1,25(OH)_2_D, suppressing Th cell proliferation through decreased Th1 cytokine production of these cells [51,52,53].

In the first instance, actions such as this would not seem to be beneficial for the genesis of allergic diseases. However, it needs to be considered that the immunosuppression of Th1 is not associated with an increase in pro-inflammatory Th2 cytokine production [54,55]. Indeed, an Austrian study showed that in cluster of differentiation (CD)4^+^ cord blood (CB) cells, 1,25(OH)_2_D_3_ suppressed Th2-driven IL-4 and IL-13 expression. Hence, 1,25(OH)_2_D_3_ induced a T-cell population without further predominance of Th2-related cytokines. It was concluded by the authors that predominantly naïve cells have a balanced effect on cytokine production, inhibiting both Th1 and Th2 cytokines [55]. 

### 5.2. Immunomodulatory Function of Vitamin D on T Regulatory Cells and Allergic Disease Development

Another group of T cells known to be potentially induced by 1,25 (OH)_2_D_3_ are regulatory T cells (Treg) [56]. Allergic disease reflects a failure to develop immunotolerance and although a part of the Th family, Treg cells act to *suppress* immune responses by other T cells and are essential in controlling inflammation and promoting tolerance to allergens.

The majority of Treg arise in the thymus. These CD4^+^FOXP3^+^ natural regulatory T cells (nTreg) mediate tolerance to self-antigens [57]. A second population of CD4^+^FOXP3^+^ Treg develop in peripheral lymphoid tissues from naïve conventional CD4^+^FOXP3^+^ T cells after exposure to antigens in combination with Transforming Growth Factor (TGF)-beta [58]. These cells are called induced regulatory T cells (iTreg) and are predominantly found within environmental interfaces such as lung respiratory mucosa and the intestines during chronic inflammation activities against microbial agents or importantly environmental (e.g., food and airborne) allergens [59,60]. Both subsets, nTreg and iTreg, play a key role in maintaining peripheral tolerance (reviewed in [61]).

Due to the strong associations between Treg and allergic diseases [62,63,64,65], identification of factors which may influence the number and function of Treg is crucial. Interestingly, vitamin D appears to influence Treg activity and differentiation [66], suggesting an influence on allergic disease and asthma outcome [66,67].

There is considerable evidence in animal studies that vitamin D3 stimulates dendritic cells (DCs), which in turn may induce IL-10-producing CD4^+^ T cells and antigen-specific Treg [68]. High levels of 1,25(OH)_2_D have been shown to induce the lineage-specific FOXP3 transcription factor, which is essential for the development and functioning of Treg [69,70] by enhancing the number and activity of circulating CD4^+^ Treg and their anti-inflammatory functions [71,72,73]. A recent study by Gorman et al. found that topical application of vitamin D in mice suppressed skin swelling in response to mechanisms that may be dependent on mast cells and Treg [74].

These animal or in vitro findings are supported by different observational studies in humans showing that higher 25(OH)D levels are associated with higher Treg/total T-cell ratios and a more immunosuppressive phenotype [75,76,77]. Furthermore, it was found that high doses of oral vitamin D influenced the functional capacity of Treg to suppress effector cells in patients with autoimmune or inflammatory disease [78,79]. 

### 5.3. Antigen-Presenting Cells and Dendritic Cells 

Antigen-presenting cells (APCs) play a significant role in the Th1/Th2 paradigm of autoimmune and allergic disease [80] and allergic disease development [81,82]. As on many other cell types, the VDR is expressed on APCs. However, beyond this DC [83], monocytes and macrophages [84] can produce 25(OH)D3 with subsequent local effects. 

Different DC subsets have been identified on sites which are frequently involved in allergic reactions such as the skin, and the respiratory and gastrointestinal tracts. For at least a decade, vitamin D3 has been suggested to program DCs for tolerance by reducing their capability to activate and generate T cells, while increasing their potential to upregulate Treg and altering receptor expression (reviewed in [85]). Treatment of DCs with 1,25(OH)D3 showed decreased production of pro-inflammatory cytokines (e.g., interferon (IFN)-α and IL-12) and increased production of the anti-inflammatory cytokine IL-10 [86]. 

In addition to being targets of vitamin D3, DCs can generate 1,25(OH)D3 locally to influence T-cell programming (reviewed in [85]). The primary function of DCs is to initiate and refine adaptive immune responses, highlighting them as a potential therapeutic tool in diseases with skewed T-cell responses, including allergic diseases. Hence, adequate 25(OH)D3 levels through preventing/correcting vitamin D deficiency may facilitate a tolerogenic, anti-inflammatory immune profile.

## 6. Studies of Geographical Variation and Allergy Outcome

Ecological studies were the first to report an inverse correlation between latitude (as a proxy of UV light exposure and vitamin D status) and allergic diseases when higher rates of allergic disease were observed at higher latitudes [87,88,89]. 

Camargo et al. reported that the distribution of adrenaline autoinjectors in the USA was greater in the northeast (8–12 prescriptions/1000 people) than the southwest (2–3 prescriptions/1000 people) [90]. Findings of this study regarding the strong latitude gradient [91] were reproduced and extended in a similar study in Australia, where the investigators found lower rates for anaphylaxis admissions and adrenaline autoinjector prescriptions with closer proximity to the equator [92]. This novel finding raised the possibility of an etiologic role for sunlight/vitamin D status particularly in food allergy, a major driver of adrenaline autoinjector prescriptions [93].

Two further studies located in the USA and South America directly associated anaphylaxis to latitude and showed a significant association [89,94]. Studies in South Korea [95] and Australia [96] focusing specifically on food-related anaphylaxis or allergy reported similar associations. Notably, the Australian study found an even stronger association between latitude and eczema [96]. Similarly a study in 12 European countries (the International Study of Asthma and Allergies in Childhood (ISAAC) study) identified that the prevalence of eczema symptoms increased with latitude and decreased with mean annual temperature [87].

Asthma outcome data from Brazil [97], the US and Australia [88] have also shown an association between latitude and asthma prevalence, further supporting the concept of a possible influence of an increasing geographical latitude on subsequently decreasing sun exposure and reduced vitamin D synthesis on allergic disease outcome. It can be hypothesized that this flow-on effect from eczema and food allergy in the earlier years to asthma in the later years may be related to the similar mechanistic immune effects underlying all allergic diseases, which are influenced by latitude as a proxy for sunlight exposure and vitamin D status. 

The strength of these geographical studies is related to the large population-based samples and the number of risk-modifying factors that can be examined. However, allergic disease outcomes are often self- (or parent-) reported or rely on correct coding of diagnosis in the hospital, which is often performed by clerical staff with restricted medical knowledge and hence prone to misclassification. Secondly, these studies are limited by different and broad geographical classifications (continent versus state, versus country, versus city). Thirdly, although associations between latitude, UV light exposure and vitamin D status are well known, many other climatic factors could also have an influence and measures of exposure are only a proxy based on the average in the population. Living in a sunny environment of lower latitude does not necessarily lead to more sun exposure (if most time is spent indoors and other sun-protective behaviour is applied) and subsequently vitamin D production [98,99,100].

This initial epidemiological evidence has since driven further observational studies and interventional trials to investigate the influence of vitamin D status on allergy prevention. Accordingly, the next sections of this literature review will explore the evidence to date from observational studies on vitamin D status and interventional randomized controlled trials (RCT).

## 7. In Utero Effects of Vitamin D Exposure and the Risk of Allergic Disease in Offspring

### 7.1. Observational Studies of Estimated Maternal Vitamin D Intake 

Numerous observational prospective cohort studies have used *maternal vitamin D intake* as an indicator of in utero vitamin D exposure and associated them to allergic disease outcomes. A self-reported food frequency questionnaire (FFQ) methodology was used to estimate maternal vitamin D intake. Study results using this methodology have been very heterogeneous, which was not surprising given that corresponding UVR exposure and associated vitamin D production were not factored in. 

For eczema outcomes, findings differed between an increased risk [101], no associations [102,103], or reduced risk [104] with higher estimated maternal vitamin D intakes. However, the results of these four studies were difficult to compare, as different dietary questionnaires and assessment tools were used; and, dependent on the study design, eczema was assessed in a wide range of age groups (at 16 to 24 months [104] vs. 3 years [102] vs. 5 years [103] of age) although it is known that eczema presents very early in childhood and is often fading at preschool age in most cases.

Compared to eczema, findings were more consistent regarding allergic respiratory diseases. Numerous cohorts have reported a reduced rate of asthma [103,105] or wheeze [102,104,106] in their offspring when food-derived maternal vitamin D intake or supplementation [107] was higher or beyond a certain cut off (reviewed in [108]). Again, generally, study designs differed significantly and some of the associations were very weak and even disappeared after adjustment for confounding factors [105], while others could not find any associations between maternal vitamin D intake and asthma or wheeze outcomes in offspring [109]. 

Two other studies performed assessments for asthma/wheeze longitudinally at two or three time points. They found a protective association with asthma but only for a limited period of time, with increasing age in the Danish study [105], but decreasing age in the study from the UK [110,111,112]. These differences may be explained by the dissimilar age groups when the allergic disease outcome assessments were conducted (at 18 months and 7 years in the Danish study vs. 5, 10 and 15 years in the UK-based study). 

A meta-analysis [112] on this topic has found that antenatal estimated vitamin D intake may have an association with childhood asthma or wheeze in children > 5 years of age. However, this meta-analysis [112] did not find any associations when examining the pooled results of maternal 25(OH)D levels, indicating that parallel longitudinal measurements of maternal vitamin D intake and 25(OH)D levels or—more preferably—well-designed intervention studies would be required to shed clearer light on this area of uncertainty. Conflicting results were also found when associating estimated maternal vitamin D intake with allergic rhinitis (AR), with a Danish study not finding any associations at 7 years of age [105], compared to a Finnish study which found higher vitamin D intakes were associated with reduced AR in offspring at 5 years of age [103]. These varied results may have been influenced by the Finnish study design involving the FFQ being filled out retrospectively, while FFQs were completed prospectively in the Danish study. 

In addition, some general weaknesses in the methodology of using FFQs need to be flagged, which challenge a direct comparison between the reviewed study results. Firstly, FFQ data were frequently collected on a single occasion, making it impossible to capture changes of dietary patterns which often occur in pregnancy. Secondly, data were collected at heterogeneous time points, ranging from first trimester, to second or third trimester. However, it is well known that particularly the timing in pregnancy could have an influence on immune development. Thirdly, different FFQs were used by the different cohorts. Fourthly, allergic disease outcomes were assessed in a wide range of age groups and dissimilar assessment criteria were applied. 

Furthermore, it is important to note that when eating a vitamin D-rich food, other nutrients with immunomodulatory properties are also being consumed. For example, a primary source of vitamin D is fatty fish, which also contains the immunomodulatory nutrient of n-3 long chain polyunsaturated fatty acids that are known to have protective effects against the development of allergic disease (reviewed in [108]). Supporting this concept, an analysis by Miyake et al. found that an inverse association between maternal vitamin D intakes and offspring eczema outcomes did not persist after adjusting for associated variations in n-3 long-chain polyunsaturated fatty acid consumption [104]. 

Some studies incorporated data on additional maternal vitamin D supplementation [102,103,105,106,109], while others did not [101,104], hence not including the total oral vitamin D intakes. Finally, and most importantly, as over 90% of vitamin D production can be induced by sunlight exposure, the lack of incorporation of measuring this element of vitamin D exposure in these studies is a major limitation.

In conclusion, results from the cohort studies investigating the influence of maternal vitamin D intake based on FFQs have multiple limitations, which may at least partially explain the heterogeneous findings regarding the influence of vitamin D on allergy outcomes. Measuring serum vitamin D levels to determine vitamin D status which captures both dietary (food and supplement) intakes as well as vitamin D produced from sunlight exposure is more reflective and should be used in preference to just reporting dietary intakes. A few of the following described studies in the next section have measured 25(OH)D levels both in pregnancy *and* at birth and associated them to allergic disease outcomes. Taking into account that there is a growing body of evidence regarding the timing [10] of various exposures for allergy preventative purposes [9], these studies will be reported in different sections, allowing a direct comparison with study results taken at the same time point in human development.

### 7.2. Observational Studies of Maternal Vitamin D Status 

Measuring 25(OH)D levels in pregnancy is a better reflection of vitamin D exposure to the developing fetus than using maternally reported estimated vitamin D intake based on FFQ methodology and/or vitamin D supplementation as it reflects both vitamin D intake and vitamin D produced by sunlight exposure. Newborn infant 25(OH)D plasma levels are correlated with maternal 25(OH)D levels, indicating that fetal and newborn infant vitamin D status are dependent on maternal vitamin D status and interventions in pregnancy have an influence on the fetus and newborn [113]. 

Two strategies have been used in observational studies to estimate the influence of fetal 25(OH)D exposure on health outcomes: the first by measurement of maternal 25(OH)D levels in pregnancy, and the second by measuring levels in cord blood (CB) at birth. This section will focus on the former.

Similar to aforementioned publications relating allergy outcomes to maternal vitamin D intakes, studies measuring maternal vitamin D status during pregnancy have also found very heterogeneous results regarding offspring allergic outcomes. Perhaps one of the key factors explaining this may be the inconsistent stages in pregnancy at which vitamin D status was assessed. Considering the significant developmental changes a fetus faces during pregnancy, this might be a major limitation, making it difficult to compare findings. Hence, study results will be grouped in this review and compared according to when 25(OH)D levels were taken in pregnancy (1st, 2nd or 3rd trimester). This will be followed by a general discussion to elucidate why the results might be so conflicting.

No studies were strictly performed in *early pregnancy* only. A British prospective population-based study where 25(OH)D levels were measured both early but also at any other stage in pregnancy could not find any associations with child eczema (at mean age 7.5 years) [114]. 

However, numerous studies with conflicting results collected maternal blood samples in *mid pregnancy.* A study in Detroit included 858 mother–child pairs of different ethnic backgrounds and found that 25(OH)D levels were inversely associated with eczema outcome (via parent report) between 0 and 2 years of age (OR, 0.85; 95% CI 0.75–0.96) [115]. This was supported by another study in the USA indicating that maternal 25(OH)D levels below 25 nmol/L were associated with a higher risk of eczema in early childhood (birth to 3 years) (aOR 2.74; 95% CI 1.37–5.49) [116]. However, two other studies did not find any associations between severely deficient, deficient and sufficient vitamin D groups and eczema outcome at 2 years of age [117] and up to 4 years of age [118]. The former study also assessed the children for food allergy and aeroallergen sensitisation at 2 years and asthma at 5 years of age and could not find any associations [117]. 

No associations between asthma/wheeze and maternal 25(OH)D levels were found in Norwegian (current asthma at 3 years of age) and Spanish (wheeze at 1 and 4 years of age and asthma at 4 to 6 years of age) studies [119,120]. Interestingly, a Danish cohort study demonstrated a U-shaped relationship, indicating that maternal vitamin D prediction scores of ≥100 and <25–30 nmol/L were associated with increased risks of “asthma” at 18 months of age [121], whereas an Australian study reported an increased risk of current wheeze at 6 years of age only in children born to mothers who were vitamin D deficient, but not by 14 years of age [122]. These heterogeneous results in *mid pregnancy* related to inconsistent asthma and wheeze outcome definitions across childhood. 

Seven studies have conducted maternal 25(OH)D measurements in *late pregnancy* (24–42 weeks of gestation) [109,123,124,125,126,127,128]. These include a German study (*n* = 378), which found that higher maternal 25(OH)D levels at 34 weeks gestation were associated with an increased risk of food allergy and allergen sensitisation within the first 2 years of life [123]. Two recent studies from Malaysia (*n* = 380) [126] and Singapore (*n* = 925) [127] examined maternal vitamin D deficiency (<30 and <50 nmol/L, respectively) and could not find any associations with food allergy in infancy [126] or food sensitisation up to 5 years of age [127].

Examining eczema outcomes in these various studies also reveals conflicting results. The aforementioned German study did not find any associations between maternal 25(OH)D levels and eczema outcomes in children within the first two years of life [123]. This finding was supported by the abovementioned Malaysian [126] and Singaporean studies [127]. A small Taiwanese cohort study (*n* = 164) found that *higher* maternal 25(OH)D levels appeared to be associated with a significantly *lower risk* of eczema (OR 0.12; 95% CI 0.02–0.63; *p* = 0.012) [129]. This differed from a larger study in the UK (*n* = 860), where *higher* maternal 25(OH)D levels were associated with a *higher risk* of eczema (adjusted OR 3.26, 95% CI 1.15–9.29) [130]. 

Regarding asthma and wheeze, most studies did not find any consistent associations with maternal 25(OH)D levels in the third trimester [109,125,126,127,128]. The Taiwanese study revealed a *lower* risk of asthma (OR 0.22; 95% CI 0.06–0.92; *p* = 0.038) at age four when maternal 25(OH)D levels were higher [129], whereas a UK-based cohort study [124] found a *higher* risk of parent-reported asthma at 9 years of age when maternal 25(OH)D levels were higher (>75 nmol/L compared to <30 nmol/L) [124].

Although it was anticipated that studies based on maternal 25(OH)D *levels* rather than self-reported maternal vitamin D *intake* may have been more conclusive, results have again been heterogeneous and conflicting. This raises the critical question of why this is the case. 

A limiting factor is the once-off nature of 25(OH)D measurements to assess maternal status. Given the strong seasonal influences UVR has on vitamin D production with possibly non-linear associations, measurements would have ideally been taken longitudinally at multiple time points. Additionally, studies use different assays for 25(OH)D measurement. While some investigations utilized liquid chromatography–tandem mass spectrometry (LC–MS/MS) [120,121,125], others used different immunoassays [109,122,124,128] or other methodologies [119,129]. The utilization of divergent types of assays could have an impact on 25(OH)D measurement and subsequently influence study outcomes [131]. Furthermore, comparison of study results was limited by various assessment tools used to evaluate allergy outcomes including standardized International Study of Asthma and Allergies in Childhood (ISAAC) [132] questionnaires [109,116,129], parent report of physician diagnosis [119,127], parent report [115,128], parent report plus name of medication [120] or clinical assessment in addition to questionnaire [115,117]. Inconclusive results for wheeze and asthma may also reflect inconsistencies across studies in the definition, diagnosis and assessment of these outcomes, especially at early ages. Moreover, while some studies captured allergic outcomes in early childhood, others focused on older age groups, making a comparison of study results very difficult. Variable results may also reflect different methodologies used to assess food or aeroallergen sensitisation—some by skin prick testing (SPT) [127], while others used blood-specific IgE levels [109,126,129], or both [115]. 

These observational studies were also performed during varied seasons and in countries with different latitudes. Maternal UV exposure may reflect the season and region of the study, and maternal UV exposure could also have vitamin D-*independent* immunomodulatory effects on allergy outcome in offspring, placing major limitations on the interpretation of these findings. 

### 7.3. Observational Studies of Cord Blood 25(OH)D Levels 

Direct neonatal measures of 25(OH)D in CB at the time of birth provide a unique window and consistent time point to investigate these relationships between vitamin D status in pregnancy and allergy outcomes. Although CB 25(OH)D levels have been shown to correlate with maternal 25(OH)D levels [129,133,134], 25(OH)D levels in CB are usually lower than maternal levels [135,136,137]. Hence, it is important to review studies on CB 25(OH)D levels and allergy outcomes separately from studies on maternal vitamin D levels.

Examining eczema outcomes in association with CB 25(OH)D levels, studies again revealed heterogeneous results. Five studies did not find any associations between CB 25(OH)D levels and eczema [116,118,123,138,139], whereas three studies found lower CB 25(OH)D to be associated with a higher risk of eczema (Table 1) [140,141,142]. Here, two of the studies were located in Australia (latitude 31.9–34.9 πS) and were performed in a high-risk population (any of the parents and/or siblings diagnosed with allergic disease) [140,142]. A recent systematic review concluded that current findings revealed no robust nor consistent associations between maternal or CB 25(OH)D status on eczema outcome in infancy [143]. 

Focusing on IgE-mediated food allergies, most studies did not find any associations (Table 1) [139,140,144]. One Australian study found higher CB 25(OH)D to be associated with less IgE-mediated food allergies at 1 year of age; however, food allergy was only medically diagnosed in 1.5% of the cohort and, due to the low incidence results, should be interpreted with caution, especially as no association between CB 25(OH)D levels and IgE-mediated food allergies was found at 3 years of age [142]. A German cohort study reported an association between higher CB 25(OH)D concentrations and an increased likelihood of parent-reported food allergy by 2 years of age. It needs to be considered that this study was conducted at a higher latitude with a subsequent lower average CB concentration (median 27 nmol/L; IQR 17; 43 nmol/L). These lower average CB levels may have contributed to different findings between studies [123].

Other CB studies have focused on allergy related respiratory outcomes, with the recognized limitation that wheezing in early childhood is typically associated with viral infection and may not herald subsequent asthma [145]. Several studies revealed an inverse relationship between CB 25(OH)D levels and wheeze [139,141,146] or asthma [138], while others could not find any associations [140,144] (Table 1). The inconsistency between results in meta-analyses may be related to different eligibility criteria, searching strategies (e.g., a literature search by Song et al. [147] consisted predominantly of Chinese databases) and study selection criteria as well as inconsistency across asthma and wheeze definitions and outcomes. 

None of the studies which assessed allergic rhinitis found any associations with CB 25(OH)D levels (Table 1) [138,141,142]. When looking at sensitisation to allergens, Rothers et al. described a “U-shaped association”, with increased risk of sensitisation at both high (>100 nmol/L) and low (<50 nmol/L) levels of CB 25(OH)D [148]. A similar U-shaped effect has been observed in other epidemiological studies looking at the risk of elevated IgE concentrations [149]. Numerous other studies did not find any associations between CB 25(OH)D levels and sensitisation (Table 1) [117,138,142,144] nor did a meta-analysis by Pacheco-Gonzalez et al. [150]. 

Overall, high heterogeneity across the reviewed observational CB studies was detected (Table 1). A major contributor for the conflicting results might have been the significant range in 25(OH)D levels (Table 1). Heterogeneous findings between studies could also be caused by inconsistencies in the definition of outcome measures, different study populations (e.g., some studies selected high-risk populations [140,142,144] while others did not), study location, sample size, duration of follow-up, assays used for vitamin D level measurement, seasonal variation, and adjustment for different covariates.

In summary, there is mixed evidence from observational studies (and associated different meta-analyses) with regard to the role of prenatal vitamin D status in the offspring’s risk of allergic manifestations. Additionally, the question of causality cannot be answered by observational studies. Hence, RCTs are warranted to shed light on the possible causative and modifiable effect of fetal vitamin D status on the development of allergic disease.

### 7.4. Randomized Controlled Trials of Prenatal Vitamin D Supplementation

Three RCTs investigated the effects of maternal vitamin D supplementation during pregnancy on offspring allergic disease development, as summarized in Table 2, with the primary outcome being allergy related respiratory symptoms [151,152,153]. All three RCTs failed to find statistically significant differences between the vitamin D-supplemented and placebo groups in terms of allergy related respiratory and other allergic disease outcomes (Table 2) [151,152,153]. 

The US Vitamin D Asthma reduction Trial (VDAART) found that allergen sensitisation was lower in the higher-vitamin-D-supplemented group [153]; however, neither the Danish nor the UK study could confirm this finding [151,152]. The results may have been influenced by different methods of evaluating sensitsation with the Danish and UK studies using both SPT *and* allergen sIgE testing, whereas the US study only used sIgE testing. Focusing further on differences in study protocols, intervention products (ergocalciferol was used by Goldring et al. versus cholecalciferol in the two other studies), doses of vitamin D and intervention periods were dissimilar, as were outcome measures and assessment tools (Table 2). 

Two studies from Europe recruited their participants from one city only (Copenhagen [151] and London [152]), whereas Litonjua et al. included pregnant women from three different health care institutions in the US located at different latitudes. Latitude is known to have an impact on UVR and could potentially influence vitamin D levels and allergy outcomes independent of vitamin D supplementation. 

In addition, the ethnicity of study populations differed (Table 2). Knowing that race and ethnicity are determinants of circulating 25(OH)D levels [154,155] and can also influence allergy outcome [156,157,158], this is an important factor. Although races were equally distributed between the intervention and control groups in the US- and UK-based studies, the investigators did not explore whether vitamin D supplementation had differential effects on maternal levels of 25(OH)D or on allergy outcomes in offspring according to ethnicity. Thus, to address this issue, a secondary analysis was performed by subdividing their cohort into different races (African American versus non-African American). However, this subsequent study was also unable to reveal a significant difference between both ethnic groups [159]. This is unsurprising, as even the original study design by Litonjua et al. [160] was not powered for clinical outcomes. Hence, in the sub-analysis, numbers per group were even lower and therefore most likely insufficient to find any effects. It also needs to be considered that in the secondary analysis, the “non-African American” population still consisted of different races (Hispanic, European and others).

Apart from the different study protocols, which make a comparison between these RCTs difficult, a major limitation of all three studies is a lack of power for clinical outcomes due to insufficient sample sizes.

In addition, the studies by Goldring et al. [152] and Chawes et al. [151] were conducted in the general population, where a sample size needs to be much larger than in a high-risk population to provide sufficient power for clinical outcomes [162]. 

Some evidence that prenatal vitamin D supplementation provided protection against wheezing was provided in the US-based study (VDAART) which was strongest for the first year of life but tended to wane between the ages of 1 and 3. However, the result did not reach significance [153]. While the authors of the Danish and US studies [151,153] argue that the non-significant trial responses observed in the individual intent-to-treat analysis of each study may be due to the lack of power, they tried to overcome the problem by combining analyses of both individual trials [163]. Compared to the Danish [151] and US-based [153] studies in isolation, the combined analysis interestingly revealed a significant effect of vitamin D supplementation on the occurrence of wheeze/asthma in offspring within the first 3 years of life particularly when 25(OH)D level was >75 nmol/L (30 ng/mL) at randomization [163]. However, it needs to be considered that combining both studies is problematic, because, as aforementioned, the study design in each individual study was different (Table 2). Firstly, vitamin D supplementation was started much earlier in the US study at 10 to 18 weeks gestation compared to 24 weeks of pregnancy in the Danish study. Secondly, the supplemented doses were different. Thirdly, the study population was dissimilar in both trials, limiting the comparison and generalizability of results. Fourthly, baseline levels of 25(OH)D vary in different geographical locations which has not been considered when combining the analyses of both studies [164]. Furthermore, the definition and assessment of outcomes (usage of heterogenous criteria and questionnaires) were varied, causing difficulties in combining and comparing the different study results (Table 2).

A meta-analysis by Garcia-Larsen et al. [165] found no overall effect on the risk of early childhood recurrent wheeze or eczema results. This meta-analysis included a fourth trial [166] conducted in Auckland, New Zealand on 260 mother–infant pairs. However, the results from this additional fourth RCT by Grant et al. were from a post hoc analysis and the primary outcome was sensitisation to aeroallergens (Table 2).

As a diagnosis of asthma is difficult to establish in early childhood, both the Danish and US groups followed their children up to 6 years of age. In these most recent publications, they also could not reveal any significant influence of high-dose vitamin D supplementation on asthma outcomes including spirometric indexes [167,168]. Asthma cases were even lower than at younger ages (high supplementation group *n* = 23/274, placebo group *n* = 18/268 children in the Danish study [167]; high supplementation group *n* = 176/442, placebo group *n* = 184/439 in the US study); hence, again, these follow-up studies were underpowered for clinical outcomes [168].

In summary, the results of these RCTs suggest that further sufficiently powered well-designed RCTs using similar study protocols are still required to determine the effect of maternal vitamin D supplementation during pregnancy as an allergy prevention strategy. There is also the question as to whether the early postnatal period may be the most critical time to ensure adequate infant vitamin D status, more so than the antenatal period. Thus, the next sections of this literature review will further explore the evidence to date in the postnatal and early childhood period.

## 8. Vitamin D Intake and Status during Childhood and Allergy Outcomes

Although it is unquestionable that the antenatal period has an important influence on the developing immune system [9,169,170,171], postnatal exposures also play a critical role [9,172,173,174]. Immune and organ development (e.g., lung) [175,176] continue upon exposure to environmental influences in the postnatal period. This includes exposures to foods, pollen, air pollution, climatic factors, and importantly in the context of this review, vitamin D and UV light [177]. 

A solid body of literature links observational studies on vitamin D status in childhood to allergic disease outcomes. To explore this in detail, this literature review will firstly describe evidence regarding postnatal vitamin D intakes, followed by vitamin D status, on allergic disease outcomes in childhood.

### 8.1. Observational Studies of Vitamin D Intake or Status in Relation to Allergy Outcomes

Lactating mothers with a sufficient 25(OH)D status still have comparatively low 25(OH)D levels of approximately 25 IU/L in their breastmilk [178]. These levels can be increased by more maternal UV light exposure [179] or vitamin D supplementation [180]. Studies have shown that daily supplementation of the lactating mother with 4000 to 6000 IU/day would be required to attain infant vitamin D sufficiency (≥50 IU/L), alternatively oral vitamin D supplementation can be given directly to the infant and/or the infant can be exposed to adequate levels of UVR [180,181,182]. 

A prospective birth cohort study in Northern Europe estimated vitamin intake from breast milk, infant formula, solid foods and vitamin D supplementation using questionnaires at 5, 7 and 10 months of age and could not find any associations with eczema at 6 years of age. However, the study results were limited by the small sample size (*n* = 123) and no serum 25(OH)D levels were measured [183]. A study in the US (*n* = 389), where vitamin D intake was determined by semi-quantitative FFQs in early childhood (median 3.2 years) found that lower vitamin D intake was associated with higher odds of persistent eczema in mid-childhood (aOR 1.37 per 100 IU decrement, 95% CI 1.01, 2.09) [116].

A Swedish study on adolescents could not find an association between vitamin D intake (reported by FFQs) and eczema or asthma at 13 years of age, and this null finding also applied for 25(OH)D levels measured at 13 years of age [184]. Another large Swedish prospective birth cohort study (*n* = 4089) investigating the use of supplements found that water-soluble vitamin D and A increased the risk of developing allergic disease (by parental questionnaires) and sensitisation to food and airborne allergens in children up to 4 years of age. Surprisingly, this effect was not seen with supplementation of the same vitamins in peanut oil. However, the study had significant limitations as the authors firstly did not specify the dose of vitamin D given, secondly no measurement of 25(OH)D levels was taken, and thirdly the possible effect of vitamin A (in isolation or in combination) on the outcome remained unclear. The mechanisms behind different results when comparing water-soluble vitamin D versus vitamin D in peanut oil remained unexplained [185].

Focusing on vitamin D blood levels taken in childhood and eczema outcomes, numerous studies could not find any associations between vitamin D status and eczema [186,187,188,189], while some found an inverse relationship [190,191] or a positive association (Table 3) [192].

Similarly, conflicting results were found for 25(OH)D levels in relation to food allergy or sensitisation with no associations [187], positive associations [193,194] or inverse associations [157,189,195]. Although both Allen et al. and Molloy et al. performed their studies in Victoria, Australia, and the major outcome was food challenge-proven food allergy, results were different. No influence of vitamin D was found in the study by Molloy et al., but this result may be related to the smaller sample size (*n* = 274) compared to the study by Allen et al. (*n* = 577). While only Allen et al. found an inverse relationship between 25(OH)D levels and food allergy outcome, it needs to be highlighted that this inverse relationship was *only* applicable for infants of Australian-born parents [157]. However, this particular relationship was not investigated in the study by Molloy et al. Furthermore, while Allen et al. took 25(OH)D levels only once (at 11–15 months) and challenges were performed at 14 to 18 months of age, 25(OH)D levels were taken earlier and twice (at birth and 6 months) by Molloy et al., who also determined outcome measures earlier (at 12 months) [187] (Table 3).

Moving to studies linking vitamin D status to allergic airway disease outcomes, results have again been heterogeneous. Some studies could not show any associations between vitamin D status and asthma, AR or aeroallergen sensitisation [188,189,190]. One study found a positive association with AR [196]; and opposed to this, three other studies revealed an inverse association with wheezing/asthma and AR or sensitisation to aeroallergens [189].

Whilst all of the aforementioned reviewed studies (Table 3) did not take longitudinal 25(OH)D measurements, Hollams et al. followed a birth cohort of high risk for allergy through the first 10 years of life. The *number of times* a child was found to be deficient in vitamin D was positively associated with the risk for asthma and wheeze at age 10 years, suggesting that prevention of vitamin D deficiency throughout the first few years of life may be an important determinant of asthma risk [191]. This mirrors earlier findings of the same group, where 25(OH)D levels at age 6 years were significant predictors of subsequent atopy/asthma-associated phenotypes at age 14 years [197]. 

**Table 3:** Observational studies of vitamin D status in childhood and allergy outcome.

Reference Study Location Latitude	Study Design	Study Population	Assay for 25(OH)D	Time Point When 25(OH)D Level Was Taken	Data Collection/ Outcome Variable	Main Results	Higher 25(OH) D Level
Sharief et al. (2011) USA 37.09 πN	National Health and Nutrition Examination Survey (NHANES)	*n* = 3136 (1 to 21 years) Non-Hispanic White 61.9% General population	CIA	Any stage between 1 and 21 years of age (mean age 12.5 years)	NHANES 2005/2006 questionnaire [189] sIgE levels for aeroallergens and foods	25(OH)D levels of <37.5 nmol/L associated with peanut (OR 2.39; CI1.29–4.45), ragweed (OR 1.83; CI1.20–2.80) and oak (OR 4.75; CI 1.53–4.94) sensitization, (sIgE) (*p* < 0.01) 25(OH)D < 50 nmol/L and sIgE associated with dog, cockroach, Alternaria species, shrimp, ryegrass, Bermuda grass, birch, thistle (*p* < 0.05) No association with eczema and AR	S↓ E←→ AR←→
Heimbeck et al. (2013) Germany 51.2 πN	Survey	*n* = 9838 (0 to 17 years) Residence in Germany (including 1411 migrants) General population	CIA	Any stage between 1 and 17 years of age (no mean age given)	Parental questionnaire: “Did a physician ever diagnose eczema?”	25(OH)D level for eczema (*n* = 1364; mean = 50.6 nmol/L) higher than for control group (*n* = 8474; mean = 47.1 nmol/L) (*p* < 0.001) Inverse association between low 25 (OH)D levels and eczema (multivariant OR for 1st quartile vs. 2nd quartile): 0.76 (95% CI 0.61–0.94))	E↑
Wang et al. (2014) Hong Kong 22 πN	Case control study	*n* = 826 (0–18 years) Eczema: *n* = 498; No eczema: *n* = 328 (no eczema: general population) Chinese population Ethnicity not further specified	Enzyme Imuno-assay	Any stage between 0 and 18 years of age Mean age in eczema group 10.5 (SD 3.9) vs. no eczema 12.3 years (SD 4.1)	Medical assessment for eczema; Hanifin and Rajka criteria applied [161] Total IgE and sIgE for aeroallergens	Eczema cases (*n* = 498, mean age 10.5 years) more frequently 25(OH)D levels < 25 nmol/L than controls (*n* = 328, mean age 12.3 years) (mean 25(OH)D level (SD); 28.9 (15.3) vs. 34.2 nmol/L (14.5); *p* < 0.001) Total IgE and sensitization to aeroallergens (sIgE and SPT): not significant between groups	E↓ S←→
Berents et al.(2016) Oslo, Norway 59.9 πN	Cross-sectional study	*n* = 623, <12 months of age *n* = 388 general population (mean age 6.5 months) *n* = 235 recruited from trial on acute bronchiolitis (mean age 4.3 months) 93% European ancestry	LC–MS/MS	Between 1 and 13 months (mean age 5.1 months) and at 24 months	Caregivers interviewed by a structured questionnaire: Hanifin and Rajka criteria applied [161]	Appointment at 1 to 13 months of age: (*n* = 67 eczema, *n* = 529 no eczema; mean age 5.1 months): 25(OH)D level (mean 58.2 nmol/L) no association with eczema At 2 year appointment (*n* = 103 eczema, *n* = 346 no eczema; mean age 24.7 months): 25(OH)D level (mean 66.9 nmol/L) no association with eczema	E←→
Allen et al. (2013) Melbourne, Australia 37.81 πS	Cohort study	*n* = 577, 11 to 15 months General population 62.5% both parents born in Australia Ethnicity not provided	LC–MS/MS	11 to 15 months	Challenge-proven food allergy at 14 to 18 months (*n* = 344), sensitized but tolerant (*n* = 74) SPT (wheal size >2 mm) or sIgE to egg, sesame, peanut, cow’s milk, shrimp	Infants of Australian-born parents only: if 25(OH)D ≤ 50 nmol/L at 12 months of age: More likely to have challenge-proven allergy to egg (aOR, 3.79; 95% CI, 1.19–12.08, *p* = 0.025) and peanut (aOR, 11.51; 95% CI, 2.01–65.79; *p* = 0.006) More likely to have multiple food allergies (>2) rather than a single food allergy (aOR, 10.48; 95% CI, 1.60–68.61 vs. aOR,1.82; 95% CI, 0.38–8.77, respectively) No parent-reported eczema	FA↓ E←→
Molloy et al. (2017) Barwon, Australia 38.14 πS	Birth cohort study	274 mother–infant pairs (mean age = 41 months (24; 60)) 75.2% European ancestry General population	LC–MS/MS	At birth and at 6 months	Challenge-proven food allergy at 1 year of age Questionnaire: Eczema UK working party criteria applied [198]	No association between 25(OH)D < 50 nmol/L at birth (*n* = 105; aRR 1.25, 95% CI 0.7–2.22) or at 6 months of age (*n* = 55; aRR 0.93, 95% CI 0.41–2.14) and food allergy at 1 year of age No association between 25(OH)D < 50 nmol/L at birth (*n* = 105; aRR 0.88, 95% CI 0.54–1.43) or at 6 months of age (*n* = 55; aRR 0.84, 95% CI 0.33–2.14) and eczema within first 12 months of age	FA←→ E←→
Cairncross et al. (2016) New Zealand 35–46 πS	Cross-sectional study	*n* = 1329 (2 to 3 years, mean age 41 months) General population 70% European ancestry	LC–MS/MS	Any stage from 24 to 60 months	ISAAC questionnaire	Food allergy associated with higher mean 25(OH)D levels (56 vs. 52 nmol/L, *p* = 0.007) 25(OH)D ≥ 75 nmol/L compared to 50–74.9 nmol/L (*n* = 29): increased risk of parent-reported food allergy (OR = 2.21, 1.33–3.68, *p* = 0.002) No associations between eczema (mean 25 (OH)D level 53 vs. 52 nmol/L, *p* = 0.50) AR (52 vs. 52 nmol/L, *p* = 0.94) Asthma (52 vs. 52 nmol/L, *p* = 0.98)	FA↑ E←→ AR←→ A←→
Bener et al. (2014} Doha, Qatar 25.28 πN	Cross-sectional study	*n* = 1833 (5 to 16 years) General population Population from Qatar Ethnicity not further specified	RIA	Any time from 5 to 16 years	Questionnaire: Parent-reported physician diagnosed	25(OH)D < 50 nmol/L associated with risk for: wheezing (OR = 1.29; *p* = 0.05) asthma (OR = 2.31: *p* = 0.001) AR (OR1.59; *p* < 0.001)	W↓ A↓ AR↓
Freishtat et al. (2010) Washington, USA 47.75 πN	Cross-sectional case control study	*n* = 113 African American Asthma *n* = 92 (6 to 20 years of age, mean age 11.1 (SE 0.4)) No asthma *n* = 21 (6 to 9 years, mean age 7 years (SE 0.3)) Controls younger than cases (*p* < 0.001)	Enzyme-linked immuno- absorbant assay	For cases: Any stage from 6 to 20 years For controls: Any stage from 6 to 9 years	Cases: Physician-diagnosed asthma Controls: General population	25(OH)D < 75 nmol/L associated with more asthma cases than controls (73/85 vs. 4/21, aOR = 42 (95% CI: 4.4 to 399) 25(OH)D < 50 nmol/L associated with more asthma cases compared to controls (46/84 vs. 1/21, aOR = 20 (95% CI:1.4 to 272)	A↓
Kutlug et al. (2017) Samsun, Turkey 41.27 πN	Case control study	*n* = 200, 6–18 years of age 100 cases with AR with seasonal symptoms for at least 2 years and positive SPT to pollen 100 controls General population	LC–MS/MS	For cases: median age 12.35 years (6; 17.8) For controls median age 11.5 years (6; 17.8)	AR diagnosis by “Allergic Rhinitis and its impact on asthma (ARIA) criteria 2008”	25(OH)D level in AR group (mean 51.95, SD 16,2) significantly higher than in control group (mean 44.8, SD 16.2), (*p* = 0.002)	AR↑
Baeck et al. (2014) Seongnam-Si, Korea 37.44 πN	Cross-sectional study	*n* = 226, age 3 to 24 months High-risk population *n* = 168 diagnosed with eczema *n* = 58 suspected food allergy, no eczema	CIA	Any time from 3 to 24 months	Physician diagnosed, parent-reported questionnaire sIgE to food (milk, egg, wheat, soy)	Polysensitization group significantly lower levels of 25(OH)D than the non-sensitization (*p* = 0.001) and monosensitization (*p* = 0.023) group 25(OH)D ≤ 50 nmol/L increased the risk of sensitization to food allergens (OR 5.0; 95% CI 1.8–14.1), milk (OR10.4; 95% CI 3.3–32.7) wheat (OR 4.2; 95% CI 1.1–15.8)	S↓
Guo et al. (2020) Guangdong, China 23.38 πN	Cross-sectional study	*n* = 2642 (at 12 or 24 months of age) General population Chinese population, not further specified	CIA	At 12 months or 24 months of age	Total IgE Food and aeroallergen sensitization by sIgE	Positive association between 25(OH)D ≥ 75 nmol/L and sIgE to cow’s milk (<0.001), cat (*p* < 0.001), dog (*p* = 0.028) Inverse association between 25(OH)D < 75 nmol/L and sIgE to Der f (*p* = 0.012) No difference in total IgE	S milk↑ S cat↑ S dog↑ S Der.farinae↓ Total IgE←→
Hollams et al. (2016) Perth, Australia 31.95 πS	Cross-sectional analysis	*n* = 263 High-risk population	LC–MS/MS	At birth and at 0.5, 1, 2, 3, 4, 5, 10 years of age	Up to 5 years symptom diary: Clinical phenotyping Total and sIgE levels	Inverse association between 25(OH)D levels and sensitization at age 0.5, 2 and 3 years. No association for all other age groups Number of 25(OH)D-deficient levels on follow ups positively associated with risk for asthma/wheeze, eczema and sensitization at 10 years of age	S↓ at 0.5, 2, 3 years S←→ at 1, 4, 5, 10 years Number of f/u: A/W↓ E↓ S↓

↓: inverse association; ←→:no association; ↑:positive association; E: eczema; FA: food allergy; W: wheeze; A: asthma; AR: allergic rhinitis; S: sensitization.

In summary, consistent with prenatal observational studies on vitamin D intake or status and allergy outcome, the reviewed literature in infancy and childhood also showed variable results. Both allergy preventing and allergy promoting effects, as well as no effects, have been described. Differences in age, study population, size and risk level of allergic disease due to family history and geographical location make it difficult to compare study results. Heterogeneity in criteria used for defining allergic disease, the possibility of non-linear associations between 25(OH)D levels and outcomes, methods for measuring 25(OH)D levels (e.g., LC–MS/MS [157,187,191] versus different types of immunoassays [189,194,195]) and variables used in the statistical adjustment contribute further to these difficulties and may at least partially explain why the results of these observational studies are heterogeneous. To overcome this uncertainty, well-designed RCTs in infants and children are needed.

### 8.2. Randomized Controlled Trials of Vitamin D Supplementation in Infancy and Allergy Outcomes

Interventional studies provide evidence for causal inference; hence, well-designed RCTs are required to shed light on the above discussed inconsistent findings. 

Rosendahl et al. compared two different vitamin D supplemental doses (400 IU/day versus 1200 IU/day) given from 2 weeks to 24 months of age. However, allergy outcomes were only analysed in a secondary analysis (primary analysis investigated the effect on bone health and infection rate) [199]. The authors were unable to find an influence on food and aeroallergen sensitisation, parent-reported physician-diagnosed wheezing, food allergy or eczema at 12 months of age [200]. Cow’s milk allergy was reported more frequently in the group receiving 1200 IU/day vitamin D supplementation (*n* = 20/387) compared with the 400 IU vitamin D group (*n* = 9/377) (OR, 2.23; 95% CI, 1.00–4.96). However, this finding needs to be interpreted with caution due to small case numbers, no corresponding sIgE levels for cow’s milk and cow’s milk allergy was based on parent report [200]. Another US-based multicenter study by Hibbs et al. found that recurrent wheezing was reduced in infants in a sustained supplementation group (400 IU/day vitamin D from 0 to 6 months of adjusted age) compared with infants in a diet-limited supplementation group (difference, −10.7% (95% CI, −27.4% to −2.9%); relative risk, 0.66 (95% CI, 0.47 to 0.94)). No differences were found for asthma, eczema, food allergy, pollen or dust allergy (parent report) or sensitisation at 12 months of age [201]. Hibbs et al. [201] primarily included preterm black infants with a low birth weight (mean gestational age 33 weeks, median birth weight 1.9 kg), including a considerable number of vitamin D-deficient or -insufficient infants. This cannot be compared to the post hoc analysis by Rosendahl et al. [200] which focused on term-born, birth weight appropriate for gestational age, White, vitamin D-sufficient infants, as it is known that ethnicity [115], prematurity and birth weight [202] can have an influence on immune development. Furthermore, any of these three infant characteristics, or the interaction between them, could lead to changes in immune function and subsequently differences in wheeze outcomes. Premature infants are also prone to developing chronic lung disease, which is known to be linked to recurrent wheeze [203], and could explain the findings in the study by Hibbs et al. Additionally, starting levels of 25(OH)D, intervention doses and intervention periods were different between both studies. No information regarding intentional or unintentional vitamin D intake (or UVR exposure) in control groups was provided. In addition to the study by Hibbs et al., the Australian Vitamin D in Allergy (VITAL) study is to date the only *primary* RCT on vitamin D supplementation in infancy and allergy outcomes in early childhood [204]. In the VITAL study, the investigators randomized term, high-risk (for allergy due to family history) infants to receive either vitamin D supplementation (400 IU/d) or a placebo until 6 months of age. The primary outcome was eczema at 6 months of age and no difference between the placebo and intervention group was found. Children were followed up until 2.5 years of age, and additional outcomes such as food allergy, wheeze/asthma, allergic rhinitis and allergen sensitisation were assessed. However, no difference between the groups regarding any of the allergy outcomes was detected [205]. 

## 9. Conclusions and Future Directions

This review indicates that there are inconsistent results in observational studies regarding associations between 25(OH)D and allergy outcomes in childhood. Furthermore, to date, no sufficiently powered intervention studies have investigated the effect of vitamin D supplementation and status in the prenatal nor early postnatal period on allergic disease development in early childhood. In addition, no single intervention study has investigated the effect of combined pre- and postnatal vitamin D supplementation and status. Moreover, only a single observational study [191] and one intervention study (the VITAL study) [131,205] analysed vitamin D status longitudinally in early childhood. 

However, clear evidence exists that vitamin D has immunomodulatory properties, and reduced supply of vitamin D to immune tissues may result in an inflammatory and allergic phenotypes [75,169,206]. In particular, vitamin D is associated with the cytokine production profiles of T cells (e.g., Th1/Th2 balance) [55], and the number or function of Treg [66,206] and DCs [85,86].

Evidence also links high latitude, and consequently reduced UV light exposure, to the increased risk of development of allergic diseases in children [96,207]. Apart from the prominent role that vitamin D production and its effects may play, sunlight *independent* of vitamin D production needs to be highlighted as a potentially critical factor in allergic disease modulation as well [96,207,208,209]. A recently published human study (the VITAL study) explored whether direct UV light exposure in infancy may have a vitamin D-independent effect on allergy prevention and found that exposure to direct UV light appears to be beneficial in reducing the risk of eczema development in early childhood [210]. Further research is required to expand these results and determine optimal levels of UV light exposure while balancing potential risks.

The lack of beneficial effects of vitamin D supplementation prenatally and in infancy [200,204,205] needs to be confirmed in larger trials adequately powered to detect clinical benefits and that even may extend beyond allergy. Future vitamin D studies should also help to address unanswered questions about the optimal time to start (if indicated) and the optimal duration of supplementation. Such studies may consider critical periods in developmental trajectories of organ systems, which may differentially affect outcomes, e.g., human lung development starts at approximately 3 to 4 weeks of gestation [211] and vitamin D plays an immunomodulatory role in the development of lungs and immune system [176]. However, the earliest prenatal intervention with vitamin D supplementation was in a RCT design which was started between 10 and 18 weeks of gestation [153]. This supplementation start time point was probably already too late to induce a preventative effect in the fetus. Hence, commencement of vitamin D supplementation for allergy prevention and potentially other health benefits may need to occur earlier in pregnancy or perhaps even before conception.

As previous observational studies found associations between vitamin D status and eczema [190], or asthma and allergic rhinitis outcomes [191,212,213] later in life (Table 3), long-term follow-up of trial interventions in needed. This is supported by immunological developments which can continue to expand until sexual maturity [175,214,215].

It is also likely that other benefits (such as on musculoskeletal health) [216] may not be obvious until later in childhood or adolescence. Furthermore, it is important to understand the wider implications of vitamin D status in specific risk populations (e.g., highly pigmented skin, premature delivery, and small for gestational age), where effects of vitamin D may be more pronounced and an intervention with 400 IU/day of vitamin D supplementation in early infancy might be protective for outcomes beyond allergy development. 

Thus, there remains an urgent need for more studies, particularly designed as intervention trials, investigating the role of 25(OH)D supplementation and status on allergy prevention. 

## Figures and Tables

**Table 1:** Prospective birth cohort studies of 25(OH)D cord blood (CB) levels and allergy outcome in childhood.

Reference and Year Latitude	Study Population	25(OH)D Level	Main Results	Higher 25(OH)D Level
Camargo et al. (2011) Wellington and Christchurch, New Zealand Latitude 41–43 πS	922 mother–child pairs General population	CB Median = 44 nmol/L (IQR 29–78)	Lower CB 25(OH)D (<10 ng/mL) levels were at higher risk for wheezing at 15 mo, 3 and 5 years than higher CB levels (>30 ng/mL) No association with asthma at 5 years of age	W↓ A←→
Rothers et al. (2011) Tuscon, Arizona Latitude 32.22 πN	219 mother–child pairs General population	CB Median = 64 nmol/L (IQR 49–81)	Lower (<50 ng/mL) and higher (≥100 ng/mL) levels of CB 25(OH)D were associated with increased total IgE and aeroallergen (sIgE) sensitization at 1, 2, 3, and 5 years of age No association with AR or asthma at 5 years	S U A←→ AR←→
Jones et al. (2012) Perth, Australia Latitude 31.95 πS	231 mother–child pairs High-risk population	CB Mean = 58 nmol/L (SD ± 24.1)	Lower CB level is associated with a higher risk for eczema at 12 months No association with wheeze, allergen sensitization or IgE-mediated food allergies	E↓ W←→ S←→ FA←→
Weisse et al. (2013) Leipzig, Germany Latitude 51.4 πN	378 mother–child pairs General population	CB median = 27 nmol/L (IQR: 17–43)	Positive association between CB 25(OH)D levels with food allergy within 2nd year of life No association with eczema and sensitization	FA↑ S←→ E←→
Baiz et al. (2014) Poitiers and Nancy, France Latitude 46–48 πN	239 mother–child pairs General population	CB Mean = 44 nmol/L (IQR: 38 nmol/L)	Inverse association between CB 25(OH)D levels with early transient wheezing and eczema by age 1, 3, and 5 years No association with asthma and AR at 5 years of age	W↓ E↓ A←→ AR←→
Stelmach et al. (2015) Lodz, Poland Latitude 51.76 πN	240 mother–child pairs General population	CB median 15.8 nmol/mL (IQR 10.4–21.3)	Inverse association between CB 25(OH)D levels wheezing in first 2 years of life No association with food allergy and eczema	W↓ E←→ FA←→
Palmer et al. (2015) Adelaide, Australia Latitude 34.92 πS	270 mother–child pairs High-risk population	CB mean 57.0 nmol/L (SD ± 24.1)	Inverse association between CB 25(OH)D and eczema to 3 years, stronger association at 1 year of age. IgE-mediated food allergies at 1 year of age in 4/260 (1.5%) children. The risk of IgE-mediated food allergies at 1 year of age (but not at 3 years of age) decreased as CB 25(OH)D concentration increased. No association with asthma, allergic rhinitis or sensitization	E↓ FA↓ A←→ AR←→
Visness et al. (2015) Baltimore, Boston, New York and St Louis, USA Latitude 38–42 πN and Madison, Wisconsin Latitude 43.1 πN	435 mother–child pairs and 258 mother–child pairs Both high-risk populations	CB median 50.3 nmol/mL (range 10.5–109.2) CB median 52.8 nmol/mL (range 10.0–194.3)	No association between CB 25(OH)D and any wheeze in first year or recurrent wheeze at 3 years No association with food allergy or food or aeroallergen sensitization to 5 years old No association with wheeze in the first 3 years, of life, food or aeroallergen sensitization to 5 years old and asthma at 6 years of age	W←→ FA←→ S←→ W←→ S←→
Gazibara et al.(2015) Rotterdam, The Netherlands Latitude 51.91 πN	2407 mother–child pairs General population	CB median 40.2 nmol/L (range 11–144.9)	No association between CB 25(OH)D levels divided into tertiles (lowest tertile (30.5 nmol/L), middle tertile (≥30.5–49), and highest tertile (≥49.0) and wheeze within the first 6 years of age	W←→
Blomberg et al. (2017) Massachusetts, USA Latitude 42.41 πN	1418 mother–child pairs General population	CB mean or median level not given	No association between CB 25(OH)D levels of sufficiency, deficiency and insufficiency (divided into 6 categories) and eczema outcome within the first 7 to 8 years of life	E←→
Hennessy et al. (2018) Cork, Ireland Latitude 51.89 πN	1050 mother–child pairs General population	CB mean 35.5 nmol/L (SD ± 18.2)	No association between CB 25(OH)D levels and: Persistent eczema in the first 2 years Food allergy and food and aeroallergen sensitization at 2 years of age Asthma at 5 years of age	E←→ FA←→ S←→ A←→

↓: inverse association; ←→: no association; ↑: positive association; U = U-shaped association; E: eczema; FA: food allergy; W: wheeze; A: asthma; AR: allergic rhinitis; S: sensitization.

**Table 2:** Randomized controlled trials of prenatal intake of vitamin D and allergic outcomes in early childhood.

Reference and Year Latitude	Study Population	Intervention Groups/Doses	Intervention Period	Duration of Intervention	Follow-Up Period/Age at Assessment	How the Outcome Was Assessed	Main Results
Goldring et al. (2013) London, UK 51.5 πN RCT, prospective	180 mothers Mixed ethnic groups: 25% Asian, 26% Middle Eastern, 25% Black, 24% White General population	Maternal: Placebo, *n* = 50 800 IU/d vitD2 *n* = 56 Single oral bolus of 200.000 IU vitD3 *n* = 52	27 weeks to birth	3 months	At 3 years of age	ISAAC questionnaire Except food allergy: physician diagnosed	800 IU/day vitD_2_ vs. placebo No difference at age 3 in: -Wheeze ever (aOR 0.56; CI 0.20–1.57; *p* = 0.27) -Eczema ever (aOR 0.61; CI 0.23–1.67; *p* = 0.34) -AR (aOR 0.63; CI 0.17–2.36; *p* = 0.49) -Food allergy (aOR 4.53; CI 0.52–39.33; *p* = 0.17) Single oral bolus of 200.000 IU vitD_3_ vs. placebo No difference at age 3 in: -Wheeze ever (aOR 1.17; CI 0.44–3.10; *p* = 0.75) -Eczema ever (aOR 0.86; CI 0.32–2.28; *p* = 0.76) -AR (aOR 0.91; CI 0.20–4.11; *p* = 0.90)
Chawes et al. (2016) Brustad et al. (2019) Denmark 55 πN RCT, prospective	623 mothers >90% European General population	Maternal: Placebo and 400 IU/d vit D3 *n* = 308 2400 IU/d and 400 IU/d vitD3 *n* = 315	24 weeks to 1 week postpartum	3.5 to 4 months	Face-to-face medical follow-up visits at 1,3,6,12,18,24,30, 36 months Sensitization tested at 6 and 18 months	Persistent wheeze/asthma diagnosed by validated quanti- tative algorithm Eczema: criteria of Hanifin and Rajka [161] For sensitization: Skin Prick Test (SPT): ≥2 mm than negative control; or sIgE	2800 IU/day vs. 400 IU/day vitD3 No difference in: -Persistent wheeze (HR 0.76, CI 0.52–1.12; *p* = 0.16) at 0–3 years -Asthma (OR 0.82; CI 0.50–1.36; *p* = 0.45) at 3 years -Eczema (HR 0.90; CI 0.65–1.26; *p* = 0.55) at 0–3 years -Sensitization at 6 or 18 mo by sIgE (OR 1.55; CI 0.89–2.73; *p* = 0.13) or SPT (OR 1.24; CI 0.66–2.31; *p* = 0.51)
Litonjua et al. (2016 and 2020) Boston, USA 42.3 πN Washington, USA 47.75 πN San Diego, USA 32.71 πN RCT, prospective	876 mothers Mixed ethnic groups: 49% Black, 13% White Hispanic, 20% White non-Hispanic 18% other High risk for allergies	Maternal: 400 IU/d vitD3, *n*= 436 4400 IU/d vitD3 *n* = 440	Between 10 and 18 weeks to delivery	5 to 7.5 months	Every 3 months: phone call At 1, 2, and 3 years of age: face-to-face assessment by scientific staff	Parent report of physician diagnosis sIgE levels	4400 IU/day vs. 400 IU/day vitD3 No difference within first 3 years in: -Recurrent wheeze/asthma (HR 0.8, CI 0.6–1.0; *p* = 0.51) -Eczema with rash (HR 0.9; CI 0.7–1.2; *p* = 0.56) Sensitization (IgE): lower in the 4400 IU/d group than in the 400 IU/d group: (10.7%; CI, 9.4–12.1% vs. 12.4%, CI, 11.45–13.5%, *p* = 0.02)
Grant et al. (2016) Auckland, New Zealand 36.84 πS RCT, post hoc	260 mother–infant pairs Mixed ethnic groups: 16% European, 24% Mãori, 43% Pacific, 17% Other General population	Maternal/infant pairs Placebo/placebo *n* = 87 1000 IU/400 IU/d vitD3, *n* = 87 2000 IU/800 IU/d vitD3, *n* = 86	Maternal: 27 weeks to birth Infant: birth to 6 months	Maternal: 3 months Infant: 6 months	Interviews at: 2, 4, 6 and 18 months	Primary care records for physician diagnosis For sensitization to aeroallergens: SPT (≥3 mm than negative control) or specific IgE levels	At 18 months of age: Sensitization Results presented for placebo, lower dose, and higher dose vitD3 - sIgE levels: Der-f1 (18% vs. 10% vs. 2%) Der-f2 (14% vs. 3% vs. 2%) Der-p1 (19% vs. 14% vs. 3%) Der-p2 (12% vs. 2% vs. 3%) For all *p* < 0.05 Grass pollen: *p* = 0.74 Cat: *p* = 0.99 -SPT: House Dust Mite (9% vs. 3% vs. 0%: *p* = 0.03) Cat: *p* = 0.74 Grass pollen: *p* = 0.99 -Asthma: (11% vs. 0% vs. 4%, *p* = 0.002)

## Data Availability

Data policy is not applicable in this review. All data can be retrieved from electronic databases (PubMed, Google Scholar, Scopus, Embase, Cochrane).

## References

[1] Christie G.L., Helms P.J., Godden D.J., Ross S.J., Friend J.A., Legge J.S., Haites N.E., Douglas J.G. (1999). Asthma, wheezy bronchitis, and atopy across two generations. Am. J. Respir. Crit. Care Med..

[2] Pearce N., Ait-Khaled N., Beasley R., Mallol J., Keil U., Mitchell E., Robertson C., Group I.P.T.S. (2007). Worldwide trends in the prevalence of asthma symptoms: Phase III of the International Study of Asthma and Allergies in Childhood (ISAAC). Thorax.

[3] Koet L.B.M., Brand P.L.P. (2018). Increase in atopic sensitization rate among Dutch children with symptoms of allergic disease between 1994 and 2014. Pediatr. Allergy Immunol. Off. Publ. Eur. Soc. Pediatr. Allergy Immunol..

[4] Rappaport S.M. (2016). Genetic Factors Are Not the Major Causes of Chronic Diseases. PLoS ONE.

[5] von Hertzen L., Beutler B., Bienenstock J., Blaser M., Cani P.D., Eriksson J., Farkkila M., Haahtela T., Hanski I., Jenmalm M.C. (2015). Helsinki alert of biodiversity and health. Ann. Med..

[6] D’Amato G., Akdis C.A. (2020). Global warming, climate change, air pollution and allergies. Allergy.

[7] D’Amato G., Chong-Neto H.J., Monge Ortega O.P., Vitale C., Ansotegui I., Rosario N., Haathela T., Galan C., Pawankar R., Murrieta M. (2020). The effects of climate change on respiratory allergy and asthma induced by pollen and mold allergens. Allergy.

[8] McFadden J.P., Thyssen J.P., Basketter D.A., Puangpet P., Kimber I. (2015). T helper cell 2 immune skewing in pregnancy/early life: Chemical exposure and the development of atopic disease and allergy. Br. J. Dermatol..

[9] Prescott S.L. (2013). Early-life environmental determinants of allergic diseases and the wider pandemic of inflammatory noncommunicable diseases. J. Allergy Clin. Immunol..

[10] Hanson M., Gluckman P. (2011). Developmental origins of noncommunicable disease: Population and public health implications. Am. J. Clin. Nutr..

[11] Hanson M.A., Gluckman P.D. (2011). Developmental origins of health and disease: Moving from biological concepts to interventions and policy. Int. J. Gynaecol. Obstet..

[12] Holick M.F. (2007). Vitamin D deficiency. N. Engl. J. Med..

[13] Holick M.F. (2016). Biological Effects of Sunlight, Ultraviolet Radiation, Visible Light, Infrared Radiation and Vitamin D for Health. Anticancer. Res..

[14] Wacker M., Holick M.F. (2013). Sunlight and Vitamin D: A global perspective for health. Derm. Endocrinol..

[15] Holick M.F., Chen T.C., Lu Z., Sauter E. (2007). Vitamin D and skin physiology: A D-lightful story. J. Bone Miner. Res. Off. J. Am. Soc. Bone Miner. Res..

[16] Faurschou A., Beyer D.M., Schmedes A., Bogh M.K., Philipsen P.A., Wulf H.C. (2012). The relation between sunscreen layer thickness and vitamin D production after ultraviolet B exposure: A randomized clinical trial. Br. J. Dermatol..

[17] Wortsman J., Matsuoka L.Y., Chen T.C., Lu Z., Holick M.F. (2000). Decreased bioavailability of vitamin D in obesity. Am. J. Clin. Nutr..

[18] Matsuoka L.Y., Ide L., Wortsman J., MacLaughlin J.A., Holick M.F. (1987). Sunscreens suppress cutaneous vitamin D3 synthesis. J. Clin. Endocrinol. Metab..

[19] Holick M.F. (2017). The vitamin D deficiency pandemic: Approaches for diagnosis, treatment and prevention. Rev. Endocr. Metab. Disord..

[20] Hintzpeter B., Scheidt-Nave C., Muller M.J., Schenk L., Mensink G.B. (2008). Higher prevalence of vitamin D deficiency is associated with immigrant background among children and adolescents in Germany. J. Nutr..

[21] Clemens T.L., Adams J.S., Henderson S.L., Holick M.F. (1982). Increased skin pigment reduces the capacity of skin to synthesise vitamin D3. Lancet.

[22] Piotrowska A., Wierzbicka J., Zmijewski M.A. (2016). Vitamin D in the skin physiology and pathology. Acta Biochim. Pol..

[23] Holick M.F. (2008). Vitamin D: A D-Lightful health perspective. Nutr. Rev..

[24] Holick M.F., Chen T.C. (2008). Vitamin D deficiency: A worldwide problem with health consequences. Am. J. Clin. Nutr..

[25] Chun R.F., Peercy B.E., Orwoll E.S., Nielson C.M., Adams J.S., Hewison M. (2014). Vitamin D and DBP: The free hormone hypothesis revisited. J. Steroid Biochem. Mol. Biol..

[26] Christakos S., Ajibade D.V., Dhawan P., Fechner A.J., Mady L.J. (2010). Vitamin D: Metabolism. Endocrinol. Metab. Clin. N. Am..

[27] DeLuca H.F. (2016). Vitamin D: Historical Overview. Vitam. Horm..

[28] Holick M.F., Binkley N.C., Bischoff-Ferrari H.A., Gordon C.M., Hanley D.A., Heaney R.P., Murad M.H., Weaver C.M., Endocrine S. (2011). Evaluation, treatment, and prevention of vitamin D deficiency: An Endocrine Society clinical practice guideline. J. Clin. Endocrinol. Metab..

[29] Chun R.F., Liu P.T., Modlin R.L., Adams J.S., Hewison M. (2014). Impact of vitamin D on immune function: Lessons learned from genome-wide analysis. Front. Physiol..

[30] Cantorna M.T. (2010). Mechanisms underlying the effect of vitamin D on the immune system. Proc. Nutr. Soc..

[31] Brennan A., Katz D.R., Nunn J.D., Barker S., Hewison M., Fraher L.J., O’Riordan J.L. (1987). Dendritic cells from human tissues express receptors for the immunoregulatory vitamin D3 metabolite, dihydroxycholecalciferol. Immunology.

[32] Morgan J.W., Kouttab N., Ford D., Maizel A.L. (2000). Vitamin D-mediated gene regulation in phenotypically defined human B cell subpopulations. Endocrinology.

[33] Provvedini D.M., Tsoukas C.D., Deftos L.J., Manolagas S.C. (1983). 1,25-dihydroxyvitamin D3 receptors in human leukocytes. Science.

[34] Umar M., Sastry K.S., Al Ali F., Al-Khulaifi M., Wang E., Chouchane A.I. (2018). Vitamin D and the Pathophysiology of Inflammatory Skin Diseases. Ski. Pharmacol. Physiol..

[35] Duplancic D., Cesarik M., Poljak N.K., Radman M., Kovacic V., Radic J., Rogosic V. (2013). The influence of selective vitamin D receptor activator paricalcitol on cardiovascular system and cardiorenal protection. Clin. Interv. Aging.

[36] Egshatyan L.V., Dudinskaya E.N., Tkacheva O.N., Kashtanova D.A. (2014). The role of vitamin D in the pathogenesis of chronic non-communicable diseases. Osteoporoz I Osteopat..

[37] Bikle D.D. (2012). Vitamin D and the skin: Physiology and pathophysiology. Rev. Endocr. Metab. Disord..

[38] Poon A.H., Laprise C., Lemire M., Montpetit A., Sinnett D., Schurr E., Hudson T.J. (2004). Association of vitamin D receptor genetic variants with susceptibility to asthma and atopy. Am. J. Respir. Crit. Care Med..

[39] Valdivielso J.M., Fernandez E. (2006). Vitamin D receptor polymorphisms and diseases. Clin. Chim. Acta Int. J. Clin. Chem..

[40] Tizaoui K., Berraies A., Hamdi B., Kaabachi W., Hamzaoui K., Hamzaoui A. (2014). Association of vitamin D receptor gene polymorphisms with asthma risk: Systematic review and updated meta-analysis of case-control studies. Lung.

[41] Wjst M. (2005). Variants in the vitamin D receptor gene and asthma. BMC Genet..

[42] Vollmert C., Illig T., Altmuller J., Klugbauer S., Loesgen S., Dumitrescu L., Wjst M. (2004). Single nucleotide polymorphism screening and association analysis--exclusion of integrin beta 7 and vitamin D receptor (chromosome 12q) as candidate genes for asthma. Clin. Exp. Allergy J. Br. Soc. Allergy Clin. Immunol..

[43] Tamasauskiene L., Golubickaite I., Ugenskiene R., Sjakste N., Paramonova N., Wu L.S., Wang L.S., Sitkauskiene B. (2021). Vitamin D receptor gene polymorphisms in atopy. Immun. Inflamm. Dis..

[44] Pillai D.K., Iqbal S.F., Benton A.S., Lerner J., Wiles A., Foerster M., Ozedirne T., Holbrook H.P., Payne P.W., Gordish-Dressman H. (2011). Associations between genetic variants in vitamin D metabolism and asthma characteristics in young African Americans: A pilot study. J. Investig. Med..

[45] Wjst M. (2006). The vitamin D slant on allergy. Pediatr. Allergy Immunol. Off. Publ. Eur. Soc. Pediatr. Allergy Immunol..

[46] Chun R.F., Peercy B.E., Adams J.S., Hewison M. (2012). Vitamin D binding protein and monocyte response to 25-hydroxyvitamin D and 1, 25-dihydroxyvitamin D: Analysis by mathematical modeling. PLoS ONE.

[47] Malik S., Fu L., Juras D.J., Karmali M., Wong B.Y., Gozdzik A., Cole D.E. (2013). Common variants of the vitamin D binding protein gene and adverse health outcomes. Crit. Rev. Clin. Lab. Sci..

[48] Carpenter T.O., Zhang J.H., Parra E., Ellis B.K., Simpson C., Lee W.M., Balko J., Fu L., Wong B.Y., Cole D.E. (2013). Vitamin D binding protein is a key determinant of 25-hydroxyvitamin D levels in infants and toddlers. J. Bone Miner. Res. Off. J. Am. Soc. Bone Miner. Res..

[49] Navas-Nazario A., Li F.Y., Shabanova V., Weiss P., Cole D.E., Carpenter T.O., Bazzy-Asaad A. (2014). Effect of vitamin D-binding protein genotype on the development of asthma in children. Ann. Allergy Asthma Immunol. Off. Publ. Am. Coll. Allergy Asthma Immunol..

[50] Tsoukas C.D., Provvedini D.M., Manolagas S.C. (1984). 1,25-dihydroxyvitamin D3: A novel immunoregulatory hormone. Science.

[51] Lemire J.M., Adams J.S., Kermani-Arab V., Bakke A.C., Sakai R., Jordan S.C. (1985). 1,25-Dihydroxyvitamin D3 suppresses human T helper/inducer lymphocyte activity in vitro. J. Immunol..

[52] Lemire J.M., Archer D.C., Beck L., Spiegelberg H.L. (1995). Immunosuppressive actions of 1,25-dihydroxyvitamin D3: Preferential inhibition of Th1 functions. J. Nutr..

[53] Jirapongsananuruk O., Melamed I., Leung D.Y. (2000). Additive immunosuppressive effects of 1,25-dihydroxyvitamin D3 and corticosteroids on TH1, but not TH2, responses. J. Allergy Clin. Immunol..

[54] Rausch-Fan X., Leutmezer F., Willheim M., Spittler A., Bohle B., Ebner C., Jensen-Jarolim E., Boltz-Nitulescu G. (2002). Regulation of cytokine production in human peripheral blood mononuclear cells and allergen-specific th cell clones by 1alpha,25-dihydroxyvitamin D3. Int. Arch. Allergy Immunol..

[55] Pichler J., Gerstmayr M., Szepfalusi Z., Urbanek R., Peterlik M., Willheim M. (2002). 1 alpha,25(OH)2D3 inhibits not only Th1 but also Th2 differentiation in human cord blood T cells. Pediatr. Res..

[56] Fisher S.A., Rahimzadeh M., Brierley C., Gration B., Doree C., Kimber C.E., Plaza Cajide A., Lamikanra A.A., Roberts D.J. (2019). The role of vitamin D in increasing circulating T regulatory cell numbers and modulating T regulatory cell phenotypes in patients with inflammatory disease or in healthy volunteers: A systematic review. PLoS ONE.

[57] Lio C.W., Hsieh C.S. (2011). Becoming self-aware: The thymic education of regulatory T cells. Curr. Opin. Immunol..

[58] Mucida D., Kutchukhidze N., Erazo A., Russo M., Lafaille J.J., Curotto de Lafaille M.A. (2005). Oral tolerance in the absence of naturally occurring Tregs. J. Clin. Investig..

[59] Kretschmer K., Apostolou I., Hawiger D., Khazaie K., Nussenzweig M.C., von Boehmer H. (2005). Inducing and expanding regulatory T cell populations by foreign antigen. Nat. Immunol..

[60] Curotto de Lafaille M.A., Kutchukhidze N., Shen S., Ding Y., Yee H., Lafaille J.J. (2008). Adaptive Foxp3+ regulatory T cell-dependent and -independent control of allergic inflammation. Immunity.

[61] Noval Rivas M., Chatila T.A. (2016). Regulatory T cells in allergic diseases. J. Allergy Clin. Immunol..

[62] Noval Rivas M., Burton O.T., Wise P., Charbonnier L.M., Georgiev P., Oettgen H.C., Rachid R., Chatila T.A. (2015). Regulatory T cell reprogramming toward a Th2-cell-like lineage impairs oral tolerance and promotes food allergy. Immunity.

[63] Syed A., Garcia M.A., Lyu S.C., Bucayu R., Kohli A., Ishida S., Berglund J.P., Tsai M., Maecker H., O’Riordan G. (2014). Peanut oral immunotherapy results in increased antigen-induced regulatory T-cell function and hypomethylation of forkhead box protein 3 (FOXP3). J. Allergy Clin. Immunol..

[64] Shreffler W.G., Wanich N., Moloney M., Nowak-Wegrzyn A., Sampson H.A. (2009). Association of allergen-specific regulatory T cells with the onset of clinical tolerance to milk protein. J. Allergy Clin. Immunol..

[65] Lloyd C.M., Hawrylowicz C.M. (2009). Regulatory T cells in asthma. Immunity.

[66] Dimeloe S., Nanzer A., Ryanna K., Hawrylowicz C. (2010). Regulatory T cells, inflammation and the allergic response—The role of glucocorticoids and Vitamin D. J. Steroid Biochem. Mol. Biol..

[67] Hartmann B., Riedel R., Jorss K., Loddenkemper C., Steinmeyer A., Zugel U., Babina M., Radbruch A., Worm M. (2012). Vitamin D receptor activation improves allergen-triggered eczema in mice. J. Investig. Dermatol..

[68] Gorman S., Judge M.A., Hart P.H. (2010). Topical 1,25-dihydroxyvitamin D3 subverts the priming ability of draining lymph node dendritic cells. Immunology.

[69] Joshi S., Pantalena L.C., Liu X.K., Gaffen S.L., Liu H., Rohowsky-Kochan C., Ichiyama K., Yoshimura A., Steinman L., Christakos S. (2011). 1,25-dihydroxyvitamin D3 ameliorates Th17 autoimmunity via transcriptional modulation of interleukin-17A. Mol. Cell. Biol..

[70] Jeffery L.E., Burke F., Mura M., Zheng Y., Qureshi O.S., Hewison M., Walker L.S., Lammas D.A., Raza K., Sansom D.M. (2009). 1,25-Dihydroxyvitamin D3 and IL-2 combine to inhibit T cell production of inflammatory cytokines and promote development of regulatory T cells expressing CTLA-4 and FoxP3. J. Immunol..

[71] Christakos S., Dhawan P., Verstuyf A., Verlinden L., Carmeliet G. (2016). Vitamin D: Metabolism, Molecular Mechanism of Action, and Pleiotropic Effects. Physiol. Rev..

[72] van Halteren A.G., Tysma O.M., van Etten E., Mathieu C., Roep B.O. (2004). 1alpha,25-dihydroxyvitamin D3 or analogue treated dendritic cells modulate human autoreactive T cells via the selective induction of apoptosis. J. Autoimmun..

[73] Van Belle T.L., Vanherwegen A.S., Feyaerts D., De Clercq P., Verstuyf A., Korf H., Gysemans C., Mathieu C. (2014). 1,25-Dihydroxyvitamin D3 and its analog TX527 promote a stable regulatory T cell phenotype in T cells from type 1 diabetes patients. PLoS ONE.

[74] Gorman S., Geldenhuys S., Weeden C.E., Grimbaldeston M.A., Hart P.H. (2018). Investigating the roles of regulatory T cells, mast cells and interleukin-9 in the control of skin inflammation by vitamin D. Arch. Dermatol. Res..

[75] Khoo A.L., Koenen H.J., Chai L.Y., Sweep F.C., Netea M.G., van der Ven A.J., Joosten I. (2012). Seasonal variation in vitamin D3 levels is paralleled by changes in the peripheral blood human T cell compartment. PLoS ONE.

[76] Prietl B., Treiber G., Mader J.K., Hoeller E., Wolf M., Pilz S., Graninger W.B., Obermayer-Pietsch B.M., Pieber T.R. (2014). High-dose cholecalciferol supplementation significantly increases peripheral CD4(+) Tregs in healthy adults without negatively affecting the frequency of other immune cells. Eur. J. Nutr..

[77] Maalmi H., Sassi F.H., Berraies A., Ammar J., Hamzaoui K., Hamzaoui A. (2013). Association of vitamin D receptor gene polymorphisms with susceptibility to asthma in Tunisian children: A case control study. Hum. Immunol..

[78] Treiber G., Prietl B., Frohlich-Reiterer E., Lechner E., Ribitsch A., Fritsch M., Rami-Merhar B., Steigleder-Schweiger C., Graninger W., Borkenstein M. (2015). Cholecalciferol supplementation improves suppressive capacity of regulatory T-cells in young patients with new-onset type 1 diabetes mellitus—A randomized clinical trial. Clin. Immunol..

[79] Piantoni S., Andreoli L., Scarsi M., Zanola A., Dall’Ara F., Pizzorni C., Cutolo M., Airo P., Tincani A. (2015). Phenotype modifications of T-cells and their shift toward a Th2 response in patients with systemic lupus erythematosus supplemented with different monthly regimens of vitamin D. Lupus.

[80] Gutcher I., Becher B. (2007). APC-derived cytokines and T cell polarization in autoimmune inflammation. J. Clin. Investig..

[81] Upham J.W., Zhang G., Rate A., Yerkovich S.T., Kusel M., Sly P.D., Holt P.G. (2009). Plasmacytoid dendritic cells during infancy are inversely associated with childhood respiratory tract infections and wheezing. J. Allergy Clin. Immunol..

[82] Silver E., Yin-DeClue H., Schechtman K.B., Grayson M.H., Bacharier L.B., Castro M. (2009). Lower levels of plasmacytoid dendritic cells in peripheral blood are associated with a diagnosis of asthma 6 yr after severe respiratory syncytial virus bronchiolitis. Pediatr. Allergy Immunol. Off. Publ. Eur. Soc. Pediatr. Allergy Immunol..

[83] Hewison M., Freeman L., Hughes S.V., Evans K.N., Bland R., Eliopoulos A.G., Kilby M.D., Moss P.A., Chakraverty R. (2003). Differential regulation of vitamin D receptor and its ligand in human monocyte-derived dendritic cells. J. Immunol..

[84] Koeffler H.P., Reichel H., Bishop J.E., Norman A.W. (1985). gamma-Interferon stimulates production of 1,25-dihydroxyvitamin D3 by normal human macrophages. Biochem. Biophys. Res. Commun..

[85] Bscheider M., Butcher E.C. (2016). Vitamin D immunoregulation through dendritic cells. Immunology.

[86] Colotta F., Jansson B., Bonelli F. (2017). Modulation of inflammatory and immune responses by vitamin D. J. Autoimmun..

[87] Weiland S.K., Husing A., Strachan D.P., Rzehak P., Pearce N., ISAAC Phase One Study Group (2004). Climate and the prevalence of symptoms of asthma, allergic rhinitis, and atopic eczema in children. Occup. Environ. Med..

[88] Krstic G. (2011). Asthma prevalence associated with geographical latitude and regional insolation in the United States of America and Australia. PLoS ONE.

[89] Hoyos-Bachiloglu R., Morales P.S., Cerda J., Talesnik E., Gonzalez G., Camargo C.A., Borzutzky A. (2014). Higher latitude and lower solar radiation influence on anaphylaxis in Chilean children. Pediatr. Allergy Immunol. Off. Publ. Eur. Soc. Pediatr. Allergy Immunol..

[90] Camargo J.C.A., Clark S., Kaplan M.S., Lieberman P., Wood R.A. (2007). Regional differences in EpiPen prescriptions in the United States: The potential role of vitamin D. J. Allergy Clin. Immunol..

[91] Fioletov V.E., McArthur L.J., Mathews T.W., Marrett L. (2010). Estimated ultraviolet exposure levels for a sufficient vitamin D status in North America. J. Photochem. Photobiol. B Biol..

[92] Mullins R.J., Clark S., Camargo C.A. (2009). Regional variation in epinephrine autoinjector prescriptions in Australia: More evidence for the vitamin D-anaphylaxis hypothesis. Ann. Allergy Asthma Immunol. Off. Publ. Am. Coll. Allergy Asthma Immunol..

[93] Rueter K., Ta B., Bear N., Lucas M., Borland M.L., Prescott S.L. (2018). Increased Use of Adrenaline in the Management of Childhood Anaphylaxis Over the Last Decade. J. Allergy Clin. Immunol. Pract..

[94] Sheehan W.J., Graham D., Ma L., Baxi S., Phipatanakul W. (2009). Higher incidence of pediatric anaphylaxis in northern areas of the United States. J. Allergy Clin. Immunol..

[95] Kim S.H., Ban G.Y., Park H.S., Kim S.C., Ye Y.M. (2016). Regional differences in vitamin D levels and incidence of food-induced anaphylaxis in South Korea. Ann. Allergy Asthma Immunol. Off. Publ. Am. Coll. Allergy Asthma Immunol..

[96] Osborne N.J., Ukoumunne O.C., Wake M., Allen K.J. (2012). Prevalence of eczema and food allergy is associated with latitude in Australia. J. Allergy Clin. Immunol..

[97] Franco J.M.G.R., Sole D., Lucia Franca V., Brabin B., Brazilian I.G. (2009). Socio-environmental conditions and geographical variability of asthma prevalence in northeast Brazil. Allergol. Immunopathol..

[98] Hamilton S.A., McNeil R., Hollis B.W., Davis D.J., Winkler J., Cook C., Warner G., Bivens B., McShane P., Wagner C.L. (2010). Profound Vitamin D Deficiency in a Diverse Group of Women during Pregnancy Living in a Sun-Rich Environment at Latitude 32 degrees N. Int. J. Endocrinol..

[99] Kimlin M., Harrison S., Nowak M., Moore M., Brodie A., Lang C. (2007). Does a high UV environment ensure adequate vitamin D status?. J. Photochem. Photobiol. B Biol..

[100] Chakhtoura M., Rahme M., Chamoun N., El-Hajj Fuleihan G. (2018). Vitamin D in the Middle East and North Africa. Bone Rep..

[101] Miyake Y., Tanaka K., Okubo H., Sasaki S., Arakawa M. (2014). Maternal consumption of dairy products, calcium, and vitamin D during pregnancy and infantile allergic disorders. Ann. Allergy Asthma Immunol. Off. Publ. Am. Coll. Allergy Asthma Immunol..

[102] Camargo C.A., Rifas-Shiman S.L., Litonjua A.A., Rich-Edwards J.W., Weiss S.T., Gold D.R., Kleinman K., Gillman M.W. (2007). Maternal intake of vitamin D during pregnancy and risk of recurrent wheeze in children at 3 y of age. Am. J. Clin. Nutr..

[103] Erkkola M., Kaila M., Nwaru B.I., Kronberg-Kippila C., Ahonen S., Nevalainen J., Veijola R., Pekkanen J., Ilonen J., Simell O. (2009). Maternal vitamin D intake during pregnancy is inversely associated with asthma and allergic rhinitis in 5-year-old children. Clin. Exp. Allergy J. Br. Soc. Allergy Clin. Immunol..

[104] Miyake Y., Sasaki S., Tanaka K., Hirota Y. (2010). Dairy food, calcium and vitamin D intake in pregnancy, and wheeze and eczema in infants. Eur. Respir. J..

[105] Maslova E., Hansen S., Jensen C.B., Thorne-Lyman A.L., Strom M., Olsen S.F. (2013). Vitamin D intake in mid-pregnancy and child allergic disease—A prospective study in 44,825 Danish mother-child pairs. BMC Pregnancy Childbirth.

[106] Devereux G., Litonjua A.A., Turner S.W., Craig L.C., McNeill G., Martindale S., Helms P.J., Seaton A., Weiss S.T. (2007). Maternal vitamin D intake during pregnancy and early childhood wheezing. Am. J. Clin. Nutr..

[107] Anderson L.N., Chen Y., Omand J.A., Birken C.S., Parkin P.C., To T., Maguire J.L., Collaboration T.A.K. (2015). Vitamin D exposure during pregnancy, but not early childhood, is associated with risk of childhood wheezing. J. Dev. Orig. Health Dis..

[108] Rueter K., Siafarikas A., Prescott S.L., Palmer D.J. (2014). In utero and postnatal vitamin D exposure and allergy risk. Expert Opin. Drug Saf..

[109] Pike K.C., Inskip H.M., Robinson S., Lucas J.S., Cooper C., Harvey N.C., Godfrey K.M., Roberts G., Southampton Women’s Survey Study G. (2012). Maternal late-pregnancy serum 25-hydroxyvitamin D in relation to childhood wheeze and atopic outcomes. Thorax.

[110] Allan K.M., Prabhu N., Craig L.C., McNeill G., Kirby B., McLay J., Helms P.J., Ayres J.G., Seaton A., Turner S.W. (2015). Maternal vitamin D and E intakes during pregnancy are associated with asthma in children. Eur. Respir. J..

[111] Devereux G., Craig L., Seaton A., Turner S. (2019). Maternal vitamin D and E intakes in pregnancy and asthma to age 15 years: A cohort study. Pediatr. Pulmonol..

[112] Shen S.Y., Xiao W.Q., Lu J.H., Yuan M.Y., He J.R., Xia H.M., Qiu X., Cheng K.K., Lam K.B.H. (2018). Early life vitamin D status and asthma and wheeze: A systematic review and meta-analysis. BMC Pulm. Med..

[113] Lee J.M., Smith J.R., Philipp B.L., Chen T.C., Mathieu J., Holick M.F. (2007). Vitamin D deficiency in a healthy group of mothers and newborn infants. Clin. Pediatr..

[114] Wills A.K., Shaheen S.O., Granell R., Henderson A.J., Fraser W.D., Lawlor D.A. (2013). Maternal 25-hydroxyvitamin D and its association with childhood atopic outcomes and lung function. Clin. Exp. Allergy J. Br. Soc. Allergy Clin. Immunol..

[115] Wegienka G., Havstad S., Zoratti E.M., Kim H., Ownby D.R., Johnson C.C. (2015). Association between vitamin D levels and allergy-related outcomes vary by race and other factors. J. Allergy Clin. Immunol..

[116] Blomberg M., Rifas-Shiman S.L., Camargo C.A., Gold D.R., Asgari M.M., Thyssen J.P., Litonjua A.A., Gillman M.W., Oken E. (2017). Low Maternal Prenatal 25-Hydroxyvitamin D Blood Levels Are Associated with Childhood Atopic Dermatitis. J. Investig. Dermatol..

[117] Hennessy A., Hourihane J.O., Malvisi L., Irvine A.D., Kenny L.C., Murray D.M., Kiely M.E. (2018). Antenatal vitamin D exposure and childhood eczema, food allergy, asthma and allergic rhinitis at 2 and 5 years of age in the atopic disease-specific Cork BASELINE Birth Cohort Study. Allergy.

[118] Gazibara T., Elbert N.J., den Dekker H.T., de Jongste J.C., Reiss I., McGrath J.J., Eyles D.W., Burne T.H., Tiemeier H., Jaddoe V.W. (2016). Associations of maternal and fetal 25-hydroxyvitamin D levels with childhood eczema: The Generation R Study. Pediatr. Allergy Immunol. Off. Publ. Eur. Soc. Pediatr. Allergy Immunol..

[119] Morales E., Romieu I., Guerra S., Ballester F., Rebagliato M., Vioque J., Tardon A., Rodriguez Delhi C., Arranz L., Torrent M. (2012). Maternal vitamin D status in pregnancy and risk of lower respiratory tract infections, wheezing, and asthma in offspring. Epidemiology.

[120] Magnus M.C., Stene L.C., Haberg S.E., Nafstad P., Stigum H., London S.J., Nystad W. (2013). Prospective study of maternal mid-pregnancy 25-hydroxyvitamin D level and early childhood respiratory disorders. Paediatr. Perinat. Epidemiol..

[121] Maslova E., Hansen S., Thorne-Lyman A.L., Jensen C.B., Strom M., Cohen A., Nielsen N.O., Olsen S.F. (2014). Predicted vitamin D status in mid-pregnancy and child allergic disease. Pediatr. Allergy Immunol. Off. Publ. Eur. Soc. Pediatr. Allergy Immunol..

[122] Zosky G.R., Hart P.H., Whitehouse A.J., Kusel M.M., Ang W., Foong R.E., Chen L., Holt P.G., Sly P.D., Hall G.L. (2014). Vitamin D deficiency at 16 to 20 weeks’ gestation is associated with impaired lung function and asthma at 6 years of age. Ann. Am. Thorac. Soc..

[123] Weisse K., Winkler S., Hirche F., Herberth G., Hinz D., Bauer M., Roder S., Rolle-Kampczyk U., von Bergen M., Olek S. (2013). Maternal and newborn vitamin D status and its impact on food allergy development in the German LINA cohort study. Allergy.

[124] Gale C.R., Robinson S.M., Harvey N.C., Javaid M.K., Jiang B., Martyn C.N., Godfrey K.M., Cooper C., Princess Anne Hospital Study G. (2008). Maternal vitamin D status during pregnancy and child outcomes. Eur. J. Clin. Nutr..

[125] Hansen S., Maslova E., Strom M., Linneberg A., Halldorsson T.I., Granstrom C., Dahl R., Hoffmann H.J., Olsen S.F. (2015). The long-term programming effect of maternal 25-hydroxyvitamin D in pregnancy on allergic airway disease and lung function in offspring after 20 to 25 years of follow-up. J. Allergy Clin. Immunol..

[126] Woon F.C., Chin Y.S., Ismail I.H., Abdul Latiff A.H., Batterham M., Chan Y.M., On Behalf Of The Micos Research G. (2020). Maternal Vitamin D Levels during Late Pregnancy and Risk of Allergic Diseases and Sensitization during the First Year of Life-A Birth Cohort Study. Nutrients.

[127] Loo E.X.L., Tham E.H., Phang K.W., Goh A., Teoh O.H., Chong Y.S., Gluckman P.D., Godfrey K.M., Van Bever H., Lee B.W. (2019). Associations between maternal vitamin D levels during pregnancy and allergic outcomes in the offspring in the first 5 years of life. Pediatr. Allergy Immunol. Off. Publ. Eur. Soc. Pediatr. Allergy Immunol..

[128] de Jongh R.T., Crozier S.R., D’Angelo S., Pike K.C., Roberts G., Lucas J.S., Inskip H., Godfrey K.M., Cooper C., Harvey N.C. (2014). Maternal 25-hydroxyvitamin D levels in relation to offspring respiratory symptoms and infections. Eur. Respir. J..

[129] Chiu C.Y., Huang S.Y., Peng Y.C., Tsai M.H., Hua M.C., Yao T.C., Yeh K.W., Huang J.L. (2015). Maternal vitamin D levels are inversely related to allergic sensitization and atopic diseases in early childhood. Pediatr. Allergy Immunol. Off. Publ. Eur. Soc. Pediatr. Allergy Immunol..

[130] Pike J.W., Meyer M.B. (2010). The vitamin D receptor: New paradigms for the regulation of gene expression by 1, 25-dihydroxyvitamin D3. Endocrinol. Metab. Clin. N. Am..

[131] Rueter K., Black L.J., Jones A., Bulsara M., Clarke M.W., Gamez C., Lim E.M., Palmer D.J., Prescott S.L., Siafarikas A. (2020). Analytical Bias in the Measurement of Plasma 25-Hydroxyvitamin D Concentrations in Infants. Int. J. Environ. Res. Public Health.

[132] Asher M.I., Keil U., Anderson H.R., Beasley R., Crane J., Martinez F., Mitchell E.A., Pearce N., Sibbald B., Stewart A.W. (1995). International Study of Asthma and Allergies in Childhood (ISAAC): Rationale and methods. Eur. Respir. J..

[133] Heckmatt J.Z., Peacock M., Davies A.E., McMurray J., Isherwood D.M. (1979). Plasma 25-hydroxyvitamin D in pregnant Asian women and their babies. Lancet.

[134] Cadario F., Savastio S., Pozzi E., Capelli A., Dondi E., Gatto M., Zaffaroni M., Bona G. (2013). Vitamin D status in cord blood and newborns: Ethnic differences. Ital. J. Pediatr..

[135] Grant C.C., Stewart A.W., Scragg R., Milne T., Rowden J., Ekeroma A., Wall C., Mitchell E.A., Crengle S., Trenholme A. (2014). Vitamin D during pregnancy and infancy and infant serum 25-hydroxyvitamin D concentration. Pediatrics.

[136] Wagner C.L., McNeil R.B., Johnson D.D., Hulsey T.C., Ebeling M., Robinson C., Hamilton S.A., Hollis B.W. (2013). Health characteristics and outcomes of two randomized vitamin D supplementation trials during pregnancy: A combined analysis. J. Steroid Biochem. Mol. Biol..

[137] Wang X., Jiao X., Tian Y., Zhang J., Zhang Y., Li J., Yang F., Xu M., Yu X., Shanghai Birth Cohort S. (2021). Associations between maternal vitamin D status during three trimesters and cord blood 25(OH)D concentrations in newborns: A prospective Shanghai birth cohort study. Eur. J. Nutr..

[138] Chawes B.L., Bonnelykke K., Jensen P.F., Schoos A.M., Heickendorff L., Bisgaard H. (2014). Cord Blood 25(OH)-Vitamin D Deficiency and Childhood Asthma, Allergy and Eczema: The COPSAC2000 Birth Cohort Study. PLoS ONE.

[139] Stelmach I., Majak P., Jerzynska J., Podlecka D., Stelmach W., Polanska K., Gromadzinska J., Wasowicz W., Hanke W. (2015). Cord serum 25-hydroxyvitamin D correlates with early childhood viral-induced wheezing. Respir. Med..

[140] Jones A.P., Palmer D., Zhang G., Prescott S.L. (2012). Cord blood 25-hydroxyvitamin D3 and allergic disease during infancy. Pediatrics.

[141] Baiz N., Dargent-Molina P., Wark J.D., Souberbielle J.C., Annesi-Maesano I., Group E.M.-C.C.S. (2014). Cord serum 25-hydroxyvitamin D and risk of early childhood transient wheezing and atopic dermatitis. J. Allergy Clin. Immunol..

[142] Palmer D.J., Sullivan T.R., Skeaff C.M., Smithers L.G., Makrides M., DOMInO Allergy Follow-Up Team (2015). Higher cord blood 25-hydroxyvitamin D concentrations reduce the risk of early childhood eczema: In children with a family history of allergic disease. World Allergy Organ. J..

[143] Mustapa Kamal Basha M.A., Majid H.A., Razali N., Yahya A. (2020). Risk of eczema, wheezing and respiratory tract infections in the first year of life: A systematic review of vitamin D concentrations during pregnancy and at birth. PLoS ONE.

[144] Visness C.M., Sandel M.T., O’Connor G., Gern J.E., Jaffee K.F., Wood R.A., Kattan M., Bloomberg G.R., Dresen A., Gergen P.J. (2015). Cord blood vitamin D concentrations are unrelated to atopy and wheeze in 2 diverse birth cohort studies. J. Allergy Clin. Immunol..

[145] Jartti T., Kuusipalo H., Vuorinen T., Soderlund-Venermo M., Allander T., Waris M., Hartiala J., Ruuskanen O. (2010). Allergic sensitization is associated with rhinovirus-, but not other virus-, induced wheezing in children. Pediatr. Allergy Immunol. Off. Publ. Eur. Soc. Pediatr. Allergy Immunol..

[146] Camargo C.A., Ingham T., Wickens K., Thadhani R., Silvers K.M., Epton M.J., Town G.I., Pattemore P.K., Espinola J.A., Crane J. (2011). Cord-blood 25-hydroxyvitamin D levels and risk of respiratory infection, wheezing, and asthma. Pediatrics.

[147] Song H., Yang L., Jia C. (2017). Maternal vitamin D status during pregnancy and risk of childhood asthma: A meta-analysis of prospective studies. Mol. Nutr. Food Res..

[148] Rothers J., Wright A.L., Stern D.A., Halonen M., Camargo J.C.A. (2011). Cord blood 25-hydroxyvitamin D levels are associated with aeroallergen sensitization in children from Tucson, Arizona. J. Allergy Clin. Immunol..

[149] Hypponen E., Berry D.J., Wjst M., Power C. (2009). Serum 25-hydroxyvitamin D and IgE—A significant but nonlinear relationship. Allergy.

[150] Pacheco-Gonzalez R.M., Garcia-Marcos L., Morales E. (2018). Prenatal vitamin D status and respiratory and allergic outcomes in childhood: A meta-analysis of observational studies. Pediatr. Allergy Immunol. Off. Publ. Eur. Soc. Pediatr. Allergy Immunol..

[151] Chawes B.L., Bonnelykke K., Stokholm J., Vissing N.H., Bjarnadottir E., Schoos A.M., Wolsk H.M., Pedersen T.M., Vinding R.K., Thorsteinsdottir S. (2016). Effect of Vitamin D3 Supplementation During Pregnancy on Risk of Persistent Wheeze in the Offspring: A Randomized Clinical Trial. JAMA J. Am. Med. Assoc..

[152] Goldring S.T., Griffiths C.J., Martineau A.R., Robinson S., Yu C., Poulton S., Kirkby J.C., Stocks J., Hooper R., Shaheen S.O. (2013). Prenatal vitamin d supplementation and child respiratory health: A randomised controlled trial. PLoS ONE.

[153] Litonjua A.A., Carey V.J., Laranjo N., Harshfield B.J., McElrath T.F., O’Connor G.T., Sandel M., Iverson R.E., Lee-Paritz A., Strunk R.C. (2016). Effect of Prenatal Supplementation With Vitamin D on Asthma or Recurrent Wheezing in Offspring by Age 3 Years: The VDAART Randomized Clinical Trial. JAMA J. Am. Med. Assoc..

[154] Ng K., Scott J.B., Drake B.F., Chan A.T., Hollis B.W., Chandler P.D., Bennett G.G., Giovannucci E.L., Gonzalez-Suarez E., Meyerhardt J.A. (2014). Dose response to vitamin D supplementation in African Americans: Results of a 4-arm, randomized, placebo-controlled trial. Am. J. Clin. Nutr..

[155] Bhagatwala J., Zhu H., Parikh S.J., Guo D.H., Kotak I., Huang Y., Havens R., Pham M., Afari E., Kim S. (2015). Dose and time responses of vitamin D biomarkers to monthly vitamin D3 supplementation in overweight/obese African Americans with suboptimal vitamin d status: A placebo controlled randomized clinical trial. BMC Obes..

[156] Wegienka G., Havstad S., Joseph C.L., Zoratti E., Ownby D., Woodcroft K., Johnson C.C. (2012). Racial disparities in allergic outcomes in African Americans emerge as early as age 2 years. Clin. Exp. Allergy J. Br. Soc. Allergy Clin. Immunol..

[157] Allen K.J., Koplin J.J., Ponsonby A.L., Gurrin L.C., Wake M., Vuillermin P., Martin P., Matheson M., Lowe A., Robinson M. (2013). Vitamin D insufficiency is associated with challenge-proven food allergy in infants. J. Allergy Clin. Immunol..

[158] Sitarik A., Havstad S., Kim H., Zoratti E.M., Ownby D., Johnson C.C., Wegienka G. (2020). Racial disparities in allergic outcomes persist to age 10 years in black and white children. Ann. Allergy Asthma Immunol. Off. Publ. Am. Coll. Allergy Asthma Immunol..

[159] Wolsk H.M., Harshfield B.J., Laranjo N., Carey V.J., O’Connor G., Sandel M., Strunk R.C., Bacharier L.B., Zeiger R.S., Schatz M. (2017). Vitamin D supplementation in pregnancy, prenatal 25(OH)D levels, race, and subsequent asthma or recurrent wheeze in offspring: Secondary analyses from the Vitamin D Antenatal Asthma Reduction Trial. J. Allergy Clin. Immunol..

[160] Litonjua A.A., Lange N.E., Carey V.J., Brown S., Laranjo N., Harshfield B.J., O’Connor G.T., Sandel M., Strunk R.C., Bacharier L.B. (2014). The Vitamin D Antenatal Asthma Reduction Trial (VDAART): Rationale, design, and methods of a randomized, controlled trial of vitamin D supplementation in pregnancy for the primary prevention of asthma and allergies in children. Contemp. Clin. Trials.

[161] Hanifin J., Rajka G. (1980). Diagnostic features of atopic dermatitis. Acta Derm. Venereol..

[162] Bahnson H.T., du Toit G., Lack G. (2017). Statistical Considerations of Food Allergy Prevention Studies. J. Allergy Clin. Immunol. Pract..

[163] Wolsk H.M., Chawes B.L., Litonjua A.A., Hollis B.W., Waage J., Stokholm J., Bonnelykke K., Bisgaard H., Weiss S.T. (2017). Prenatal vitamin D supplementation reduces risk of asthma/recurrent wheeze in early childhood: A combined analysis of two randomized controlled trials. PLoS ONE.

[164] Kimlin M.G., Olds W.J., Moore M.R. (2007). Location and vitamin D synthesis: Is the hypothesis validated by geophysical data?. J. Photochem. Photobiol. B Biol..

[165] Garcia-Larsen V., Ierodiakonou D., Jarrold K., Cunha S., Chivinge J., Robinson Z., Geoghegan N., Ruparelia A., Devani P., Trivella M. (2018). Diet during pregnancy and infancy and risk of allergic or autoimmune disease: A systematic review and meta-analysis. PLoS Med..

[166] Grant C.C., Crane J., Mitchell E.A., Sinclair J., Stewart A., Milne T., Knight J., Gilchrist C., Camargo J.C.A. (2016). Vitamin D supplementation during pregnancy and infancy reduces aeroallergen sensitization: A randomized controlled trial. Allergy.

[167] Brustad N., Eliasen A.U., Stokholm J., Bonnelykke K., Bisgaard H., Chawes B.L. (2019). High-Dose Vitamin D Supplementation During Pregnancy and Asthma in Offspring at the Age of 6 Years. JAMA J. Am. Med. Assoc..

[168] Litonjua A.A., Carey V.J., Laranjo N., Stubbs B.J., Mirzakhani H., O’Connor G.T., Sandel M., Beigelman A., Bacharier L.B., Zeiger R.S. (2020). Six-Year Follow-up of a Trial of Antenatal Vitamin D for Asthma Reduction. N. Engl. J. Med..

[169] Hornsby E., Pfeffer P.E., Laranjo N., Cruikshank W., Tuzova M., Litonjua A.A., Weiss S.T., Carey V.J., O’Connor G., Hawrylowicz C. (2018). Vitamin D supplementation during pregnancy: Effect on the neonatal immune system in a randomized controlled trial. J. Allergy Clin. Immunol..

[170] Gluckman P.D., Hanson M.A., Cooper C., Thornburg K.L. (2008). Effect of in utero and early-life conditions on adult health and disease. N. Engl. J. Med..

[171] McDade T.W. (2012). Early environments and the ecology of inflammation. Proc. Natl. Acad. Sci. USA.

[172] Strachan D.P. (1989). Hay fever, hygiene, and household size. Br. Med. J..

[173] Schaub B., Lauener R., von Mutius E. (2006). The many faces of the hygiene hypothesis. J. Allergy Clin. Immunol..

[174] Prescott S.L., Smith P., Tang M., Palmer D.J., Sinn J., Huntley S.J., Cormack B., Heine R.G., Gibson R.A., Makrides M. (2008). The importance of early complementary feeding in the development of oral tolerance: Concerns and controversies. Pediatr. Allergy Immunol. Off. Publ. Eur. Soc. Pediatr. Allergy Immunol..

[175] Joshi S., Kotecha S. (2007). Lung growth and development. Early Hum. Dev..

[176] Henderson A.J., Warner J.O. (2012). Fetal origins of asthma. Semin. Fetal Neonatal Med..

[177] Rueter K., Haynes A., Prescott S.L. (2015). Developing Primary Intervention Strategies to Prevent Allergic Disease. Curr. Allergy Asthma Rep..

[178] Hollis B.W., Roos B.A., Draper H.H., Lambert P.W. (1981). Vitamin D and its metabolites in human and bovine milk. J. Nutr..

[179] Greer F.R., Hollis B.W., Cripps D.J., Tsang R.C. (1984). Effects of maternal ultraviolet B irradiation on vitamin D content of human milk. J. Pediatr..

[180] Hollis B.W., Wagner C.L. (2013). Clinical review: The role of the parent compound vitamin D with respect to metabolism and function: Why clinical dose intervals can affect clinical outcomes. J. Clin. Endocrinol. Metab..

[181] Wagner C.L., Hulsey T.C., Fanning D., Ebeling M., Hollis B.W. (2006). High-dose vitamin D3 supplementation in a cohort of breastfeeding mothers and their infants: A 6-month follow-up pilot study. Breastfeed. Med. Off. J. Acad. Breastfeed. Med..

[182] Hollis B.W., Wagner C.L. (2004). Vitamin D requirements during lactation: High-dose maternal supplementation as therapy to prevent hypovitaminosis D for both the mother and the nursing infant. Am. J. Clin. Nutr..

[183] Gallo S., Comeau K., Vanstone C., Agellon S., Sharma A., Jones G., L’Abbe M., Khamessan A., Rodd C., Weiler H. (2013). Effect of different dosages of oral vitamin D supplementation on vitamin D status in healthy, breastfed infants: A randomized trial. JAMA J. Am. Med. Assoc..

[184] Barman M., Jonsson K., Hesselmar B., Sandin A., Sandberg A.S., Wold A.E. (2015). No association between allergy and current 25-hydroxy vitamin D in serum or vitamin D intake. Acta Paediatr..

[185] Kull I.B.A., Melén E., Lilja G., van Hage M., Pershagen G., Wickman M. (2006). Earlylife supplementation of vitamins A and D, in water-soluble form or in peanut oil, and allergic diseases during childhood. J. Allergy Clin. Immunol..

[186] Berents T.L., Lodrup Carlsen K.C., Mowinckel P., Sandvik L., Skjerven H.O., Rolfsjord L.B., Kvenshagen B., Hunderi J.O., Bradley M., Lieden A. (2016). Vitamin D levels and atopic eczema in infancy and early childhood in Norway: A cohort study. Br. J. Dermatol..

[187] Molloy J., Koplin J.J., Allen K.J., Tang M.L.K., Collier F., Carlin J.B., Saffery R., Burgner D., Ranganathan S., Dwyer T. (2017). Vitamin D insufficiency in the first 6 months of infancy and challenge-proven IgE-mediated food allergy at 1 year of age: A case-cohort study. Allergy.

[188] Cairncross C., Grant C., Stonehouse W., Conlon C., McDonald B., Houghton L., Eyles D., Camargo C.A., Coad J., von Hurst P. (2016). The Relationship between Vitamin D Status and Allergic Diseases in New Zealand Preschool Children. Nutrients.

[189] Sharief S., Jariwala S., Kumar J., Muntner P., Melamed M.L. (2011). Vitamin D levels and food and environmental allergies in the United States: Results from the National Health and Nutrition Examination Survey 2005–2006. J. Allergy Clin. Immunol..

[190] Wang S.S., Hon K.L., Kong A.P., Pong H.N., Wong G.W., Leung T.F. (2014). Vitamin D deficiency is associated with diagnosis and severity of childhood atopic dermatitis. Pediatr. Allergy Immunol. Off. Publ. Eur. Soc. Pediatr. Allergy Immunol..

[191] Hollams E.M., Teo S.M., Kusel M., Holt B.J., Holt K.E., Inouye M., De Klerk N.H., Zhang G., Sly P.D., Hart P.H. (2017). Vitamin D over the first decade and susceptibility to childhood allergy and asthma. J. Allergy Clin. Immunol..

[192] Heimbeck I., Wjst M., Apfelbacher C.J. (2013). Low vitamin D serum level is inversely associated with eczema in children and adolescents in Germany. Allergy.

[193] Cairncross C.T., Stonehouse W., Conlon C.A., Grant C.C., McDonald B., Houghton L.A., Eyles D., Camargo C.A., Coad J., von Hurst P.R. (2017). Predictors of vitamin D status in New Zealand preschool children. Matern. Child Nutr..

[194] Guo Y., Yu L., Deng Y.H., Ke H.J., Wu J.L. (2020). Associations between serum 25-hydroxyvitamin D levels and allergic sensitization in early childhood. Allergol. Immunopathol..

[195] Baek J.H., Shin Y.H., Chung I.H., Kim H.J., Yoo E.G., Yoon J.W., Jee H.M., Chang Y.E., Han M.Y. (2014). The Link between Serum Vitamin D Level, Sensitization to Food Allergens, and the Severity of Atopic Dermatitis in Infancy. J. Pediatr..

[196] Kutlug S., Kilic M., Bilgici B., Paksu S., Yildiran A., Sancak R. (2017). An evaluation of vitamin D levels in children with seasonal allergic rhinitis during pollen season. Pediatr. Allergy Immunol. Off. Publ. Eur. Soc. Pediatr. Allergy Immunol..

[197] Hollams E.M., Hart P.H., Holt B.J., Serralha M., Parsons F., de Klerk N.H., Zhang G., Sly P.D., Holt P.G. (2011). Vitamin D and atopy and asthma phenotypes in children: A longitudinal cohort study. Eur. Respir. J..

[198] Williams H.C., Burney P.G., Pembroke A.C., Hay R.J. (1994). The U.K. Working Party’s Diagnostic Criteria for Atopic Dermatitis. III. Independent hospital validation. Br. J. Dermatol..

[199] Rosendahl J., Valkama S., Holmlund-Suila E., Enlund-Cerullo M., Hauta-Alus H., Helve O., Hytinantti T., Levalahti E., Kajantie E., Viljakainen H. (2018). Effect of Higher vs Standard Dosage of Vitamin D3 Supplementation on Bone Strength and Infection in Healthy Infants: A Randomized Clinical Trial. JAMA Pediatr..

[200] Rosendahl J., Pelkonen A.S., Helve O., Hauta-Alus H., Holmlund-Suila E., Valkama S., Enlund-Cerullo M., Viljakainen H., Hytinantti T., Makitie O. (2019). High-Dose Vitamin D Supplementation Does Not Prevent Allergic Sensitization of Infants. J. Pediatr..

[201] Hibbs A.M., Ross K., Kerns L.A., Wagner C., Fuloria M., Groh-Wargo S., Zimmerman T., Minich N., Tatsuoka C. (2018). Effect of Vitamin D Supplementation on Recurrent Wheezing in Black Infants Who Were Born Preterm: The D-Wheeze Randomized Clinical Trial. JAMA J. Am. Med. Assoc..

[202] Goedicke-Fritz S., Hartel C., Krasteva-Christ G., Kopp M.V., Meyer S., Zemlin M. (2017). Preterm Birth Affects the Risk of Developing Immune-Mediated Diseases. Front. Immunol..

[203] Kotecha S., Clemm H., Halvorsen T., Kotecha S.J. (2018). Bronchial hyper-responsiveness in preterm-born subjects: A systematic review and meta-analysis. Pediatr. Allergy Immunol. Off. Publ. Eur. Soc. Pediatr. Allergy Immunol..

[204] Rueter K., Jones A.P., Siafarikas A., Lim E.M., Bear N., Noakes P.S., Prescott S.L., Palmer D.J. (2019). Direct infant UV light exposure is associated with eczema and immune development. J. Allergy Clin. Immunol..

[205] Rueter K., Jones A.P., Siafarikas A., Lim E.M., Prescott S.L., Palmer D.J. (2020). In “High-Risk” Infants with Sufficient Vitamin D Status at Birth, Infant Vitamin D Supplementation Had No Effect on Allergy Outcomes: A Randomized Controlled Trial. Nutrients.

[206] Maalmi H., Berraies A., Tangour E., Ammar J., Abid H., Hamzaoui K., Hamzaoui A. (2012). The impact of vitamin D deficiency on immune T cells in asthmatic children: A case-control study. J. Asthma Allergy.

[207] Mullins R.J., Camargo C.A. (2012). Latitude, sunlight, vitamin D, and childhood food allergy/anaphylaxis. Curr. Allergy Asthma Rep..

[208] Thyssen J.P., Zirwas M.J., Elias P.M. (2015). Potential role of reduced environmental UV exposure as a driver of the current epidemic of atopic dermatitis. J. Allergy Clin. Immunol..

[209] Hwang J.M., Oh S.H., Shin M.Y. (2016). The relationships among birth season, sunlight exposure during infancy, and allergic disease. Korean J. Pediatr..

[210] Rueter K., Jones A.P., Siafarikas A., Chivers P., Prescott S.L., Palmer D.J. (2021). The Influence of Sunlight Exposure and Sun Protecting Behaviours on Allergic Outcomes in Early Childhood. Int. J. Environ. Res. Public Health.

[211] Chen L., Zosky G.R. (2017). Lung development. Photochem. Photobiol. Sci. Off. J. Eur. Photochem. Assoc. Eur. Soc. Photobiol..

[212] Bener A., Ehlayel M.S., Bener H.Z., Hamid Q. (2014). The impact of Vitamin D deficiency on asthma, allergic rhinitis and wheezing in children: An emerging public health problem. J. Family Community Med..

[213] Freishtat R.J., Iqbal S.F., Pillai D.K., Klein C.J., Ryan L.M., Benton A.S., Teach S.J. (2010). High prevalence of vitamin D deficiency among inner-city African American youth with asthma in Washington, DC. J. Pediatr..

[214] Upham J.W., Lee P.T., Holt B.J., Heaton T., Prescott S.L., Sharp M.J., Sly P.D., Holt P.G. (2002). Development of interleukin-12-producing capacity throughout childhood. Infect. Immun..

[215] Nelson D.J., Holt P.G. (1995). Defective regional immunity in the respiratory tract of neonates is attributable to hyporesponsiveness of local dendritic cells to activation signals. J. Immunol..

[216] Lips P., van Schoor N.M. (2011). The effect of vitamin D on bone and osteoporosis. Best Pract. Res. Clin. Endocrinol. Metab..

